# Compartmentalized dendritic plasticity in the mouse retrosplenial cortex links contextual memories formed close in time

**DOI:** 10.1038/s41593-025-01876-8

**Published:** 2025-02-17

**Authors:** Megha Sehgal, Daniel Almeida Filho, George Kastellakis, Sungsoo Kim, Jinsu Lee, Yang Shen, Shan Huang, Ayal Lavi, Giselle Fernandes, Irene Davila Mejia, Sunaina Soans Martin, Asli Pekcan, Melody Shana Wu, Won Do Heo, Panayiota Poirazi, Joshua T. Trachtenberg, Alcino J. Silva

**Affiliations:** 1 Departments of Neurobiology, Psychiatry and Psychology & Integrative Center for Learning and Memory, University of California, Los Angeles, Los Angeles, CA USA; 2 Institute of Molecular Biology and Biotechnology (IMBB), Foundation for Research and Technology, Hellas (FORTH), Heraklion, Greece; 3 Department of Biological Sciences, Korea Advanced Institute of Science and Technology, Daejeon, Republic of Korea; 4 Present Address: Department of Psychology, The Ohio State University, Columbus, OH USA; 5 Present Address: SENAI Institute of Innovation in Advanced Health Systems, University Center SENAI CIMATEC, Salvador, Brazil

**Keywords:** Fear conditioning, Cellular neuroscience, Synaptic plasticity

## Abstract

Events occurring close in time are often linked in memory, and recent studies suggest that such memories are encoded by overlapping neuronal ensembles. However, the role of dendritic plasticity mechanisms in linking memories is unknown. Here we show that memory linking is dependent not only on neuronal ensemble overlap in the mouse retrosplenial cortex, but also on branch-specific dendritic allocation mechanisms. The same dendritic segments are preferentially activated by two linked (but not independent) contextual memories, and spine clusters added after each of two linked (but not independent) contextual memories are allocated to the same dendritic segments. Importantly, we show that the reactivation of dendrites activated during the first context exploration is sufficient to link two contextual memories. Our results demonstrate a critical role for localized dendritic plasticity in memory integration and reveal rules governing how linked and independent memories are allocated to dendritic compartments.

## Abstract (web summary)

Sehgal et al. show that branch-specific dendritic plasticity mechanisms in the retrosplenial cortex link memories acquired close in time such that, unlike independent memories, linked memories are encoded by many of the same dendritic branches.

## Main

Memory formation is a dynamic process, where single memories are stored, updated and integrated within the framework of other preexisting memories to drive adaptive behavior^1,2^. Recent studies in rodents have revealed that the overlap between the neuronal ensembles encoding different memories can link them, such that the recall of one leads to the recall of the other^3–5^. A similar process in humans is believed to mediate inferential reasoning^6^ and other forms of memory organization. Transient increases in neuronal excitability drive ensemble overlap^3,5,7^, but the neuronal locus and specific form of cellular plasticity underlying these changes are unknown.

Within the brain, pyramidal neurons use their elaborate dendritic structures to perform computations previously thought impossible for a single cell^8,9^. The molecular and cellular physiology that supports these complex computations within a single cell and how these computations influence ensemble activation, and thus animal behavior, are poorly understood. Focal synaptic activity on dendritic segments results in compartmentalized dendritic plasticity, which in turn regulates the integration and propagation of local dendritic signals to the soma, and impacts future induction of synaptic plasticity on these dendritic segments^10–14^. Although such localized plasticity within dendritic branches is likely to influence many neural processes, it is unclear whether and how this plasticity modulates memory.

Because experience-dependent dendritic plasticity is branch specific^10–12,14^, and potentiation of dendritic spines can affect future plasticity at nearby spines on the same dendritic branch^12,15^, we hypothesized that two memories acquired close in time would be allocated to an overlapping population of dendritic branches, and that this mechanism drives linking of distinct memories. We investigated the role of dendritic allocation mechanisms in contextual memory linking within the retrosplenial cortex (RSC), a brain region important for spatial and contextual memory processing^16,17^. Using activity-dependent labeling and manipulation approaches, longitudinal one-photon and two-photon imaging of somatic and dendritic compartments, and computational modeling, we show that memory linking is dependent not only on ensemble overlap but also on branch-specific dendritic allocation. Our results demonstrate an important role for localized dendritic plasticity mechanisms in the formation and integration of related memories.

## Results

### Overlap in RSC ensembles representing linked memories

**Fig. 1:**
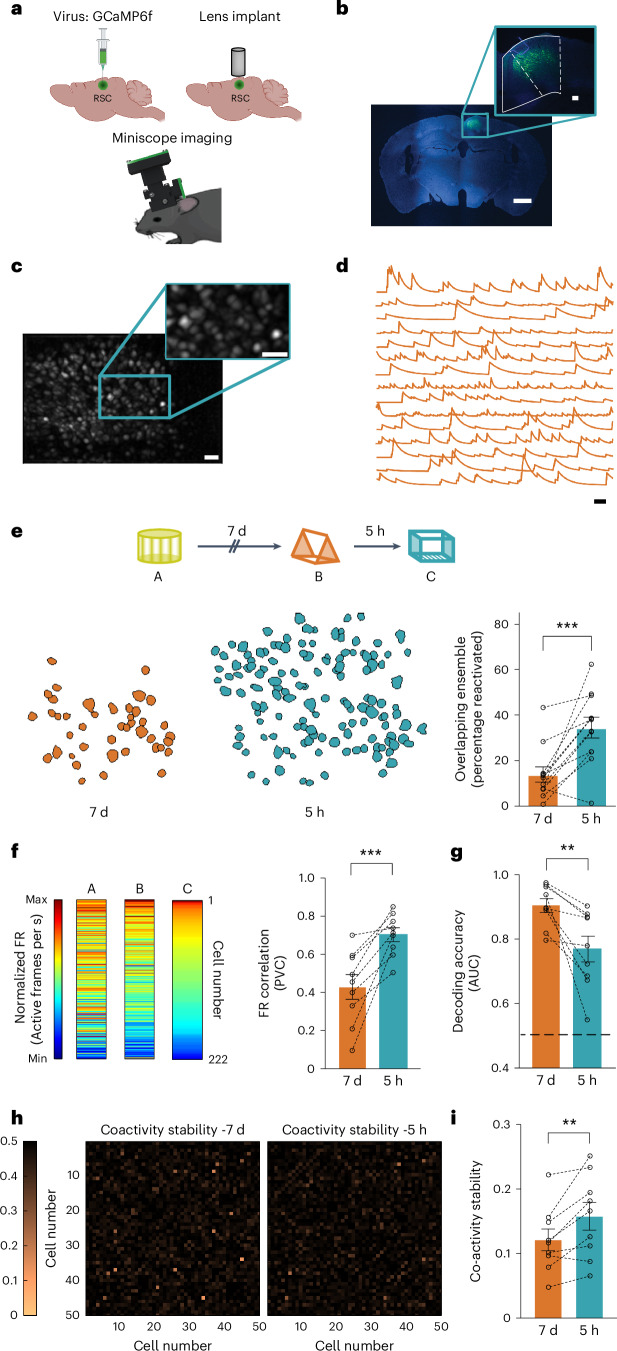
Overlapping RSC ensembles are recruited to encode contextual memories acquired close in time. **a**, Miniscope methodology. **b**, GCaMP6f expression within the RSC. Scale bars, 1 mm and 100 µm (inset). **c**, Example maximum intensity projection of processed calcium signals during context exploration. Scale bars, 50 µm and 50 µm (inset). **d**, Representative calcium traces from 15 putative RSC neurons from one mouse. Scale bar, 30 s. **e**, Overlapping RSC ensembles encode distinct memories acquired close in time. Top: mice were imaged while exploring three novel contexts (A, B and C) separated by 7 days or 5 h. Bottom left: overlapping neurons in RSC ensembles in a representative mouse when contexts were separated by 7 days and 5 h. Bottom right: RSC neuronal ensembles displayed greater overlap when contexts were separated by 5 h versus 7 days (*n* = 12 mice per group, paired *t*-test, *t* = 4.6, *P* = 0.0008). **f**, RSC neurons with a high frequency of calcium transients continued to fire at high rates when contexts were explored close in time. Left: frequency of calcium transients for all RSC neurons from one mouse (normalized to the frequency of calcium transients in context C). Right: population vector correlation (PVC) for normalized FRs (*n* = 9 mice per group; paired *t*-test, *t* = 5.1, *P* = 0.0009). **g**, An NB classifier is better at distinguishing two contexts explored 7 days versus 5 h apart. The area under the curve (AUC) for the binary NB classification was higher for sessions recorded 7 days apart (*n* = 9 mice per group; paired *t*-test, *t* = 3.5, *P* = 0.008). Dashed line indicates chance performance (AUC = 0.5). **h**, The stability of neuronal coactivity across sessions is represented as the absolute difference in PWCs between sessions. 50 cell pairs; higher numbers (darker color) indicate more stable coactivity patterns. **i**, The coactivity of neuronal pairs is more stable in two contexts explored 5 h versus 7 days apart (*n* = 9 mice per group; paired *t*-test, *t* = 3.4, *P* = 0.009). Data represent the mean ± s.e.m. and each data point. All *t*-tests were two tailed. **P < 0.01, ***P < 0.001. Source data

**Extended Data Fig. 1:**
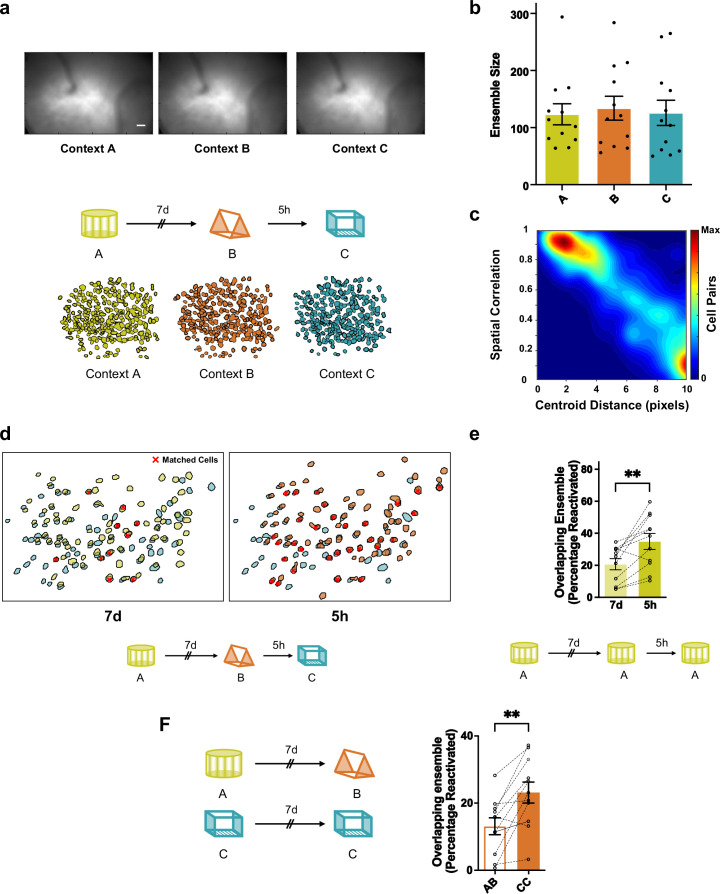
Stability of imaging and neuronal registration across days. (**a**) Top: Images of mean fluorescence from each session from a representative mouse. Scale: 50 µm. These images from each session were cross-registered with each other (see Methods). Bottom: Description of imaging paradigm and RSC ensemble segmented from a mouse. (**b**) RSC ensemble size remains stable across hours and days when different contexts are imaged (4599 putative RSC neurons, 132.9 ± 11.6 neurons per session, One-way repeated measures ANOVA, F (1.08, 11.9) = 0.52, *p* = 0.5, n = 12 mice per group). Please note that although the size of these ensembles remains unchanged, the neurons participating in these ensembles may change. (**c**) Spatial correlation and centroid distance were calculated for all cell pairs from all mice. Ensemble overlap using a range of criteria from spatial correlation ≥ 0.6-0.95 and centroid distance ≤ 3-9 pixels is shown in Supplementary Table 1. (**d**) Example cross-registration of neurons in a mouse from sessions 7 days and 5 hours apart. Red cross indicates matched cells. Cross-registration criteria: spatial correlation = 0.9 and centroid distance = 4 pixels. (**e**) Representational drift over a week: Mice were exposed to the same context (AAA) five hours or seven days apart. RSC neuronal ensembles display greater overlap when mice experience the same context 5 hours vs. 7 days apart. (n = 11 mice per group; paired t-test, t = 3.9, *p* = 0.003). (**f**) Neuronal ensemble stability over a week: Mice were exposed to two different (AB) or the same context (CC) seven days apart. All context presentations were counterbalanced. RSC neuronal ensembles display greater overlap when mice experience the same context 7 days apart vs. distinct contexts. (n = 11 mice per group, paired t-test, t = 4.07, *p* = 0.002). Data represent mean ± s.e.m. and each data point; all comparisons were two-tailed. Source data

The overlap between neuronal ensembles encoding two memories (neuronal co-allocation) is critical for linking these memories^3–5^. However, it is unclear if such neuronal overlap is observed within the RSC, a brain region critical for encoding contextual memories. Thus, we first investigated whether RSC neuronal ensembles representing memories of two contexts explored close in time (or linked memories)^3^ also display a higher overlap than two ensembles representing memories encoded further apart (or independent memories). We used a customized head-mounted miniature microscope to image calcium dynamics in RSC neurons (Fig. 1a–d and Extended Data Fig. 1; 4,599 RSC neurons, 132.9 ± 11.6 neurons per session) while mice explored different contexts. We found a greater overlap between the RSC neuronal ensembles activated during the encoding of two contexts explored 5 h apart (linked) versus 7 days apart (unlinked; Fig. 1e). Our results cannot be attributed to differences in ensemble size (Extended Data Fig. 1) or the criteria used for cross-registration across days (Supplementary Table 1). Consistent with previous results^18^, we discovered substantial representational drift in the neuronal ensemble representing the same context 7 days apart. Nevertheless, ensembles representing the same context were more stable than those representing two distinct contexts (Extended Data Fig. 1). Thus, greater overlap between the RSC neuronal ensembles representing two contexts explored 5 h versus 7 days apart is unlikely to be due to representational drift alone or problems with longitudinal imaging itself. These data indicate that RSC neurons represent temporally proximate contextual memories using overlapping neuronal populations.

**Extended Data Fig. 2:**
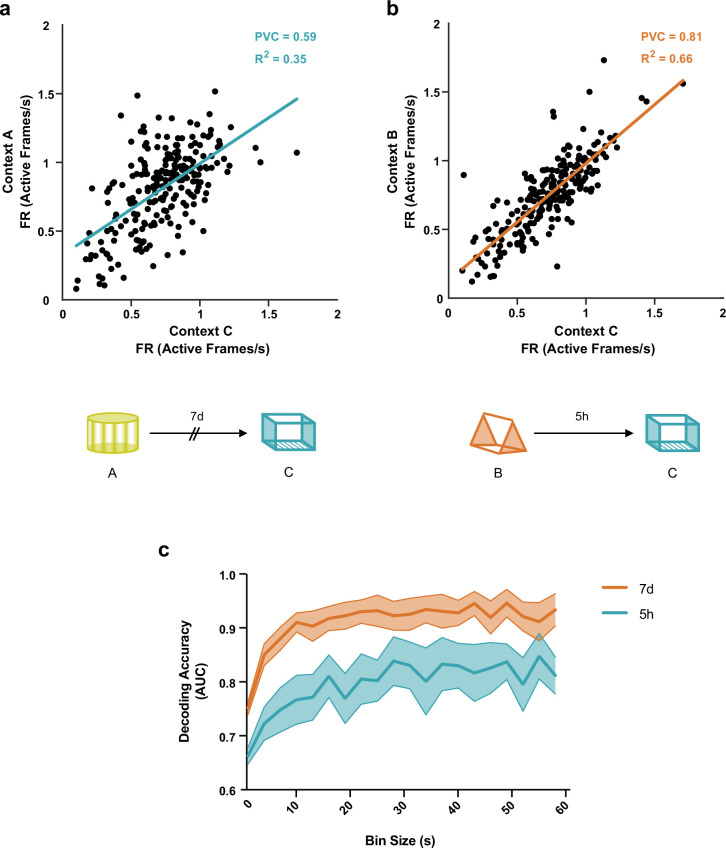
Neuronal activity is more correlated when two contexts are explored closer in time. (**a**, **b**) Scatter plot of the firing rate of all neurons from one mouse in context A (a, 7 days apart) and context B (b, 5 hours apart) as a function of firing rate in context C highlights that neuronal firing rate is maintained when two contexts are explored close in time. Lines represent least-squares linear regression. (**c**) Naive Bayes (NB) classifier is more accurate at distinguishing imaging sessions recorded 7 days vs. 5 hours apart irrespective of bin size. AUC (area under the curve) for the binary NB classification between sessions recorded 7 days (7d) apart or 5 hours (5h) apart using neuronal activity indicates that neuronal activity can be used to distinguish between contexts explored 7 days apart more accurately than contexts explored 5 hours apart. Spike probabilities were binned for non-overlapping intervals ranging from 0.5 to 60 seconds (step size 0.5 s; TWRM ANOVA for AUC by bin size; F_Group_ (1, 16) = 6.2, *p* = 0.02, n = 9 mice per group). Data represent mean ± s.e.m. Chance Levels performance of the AUC = 0.5. Source data

**Extended Data Fig. 3:**
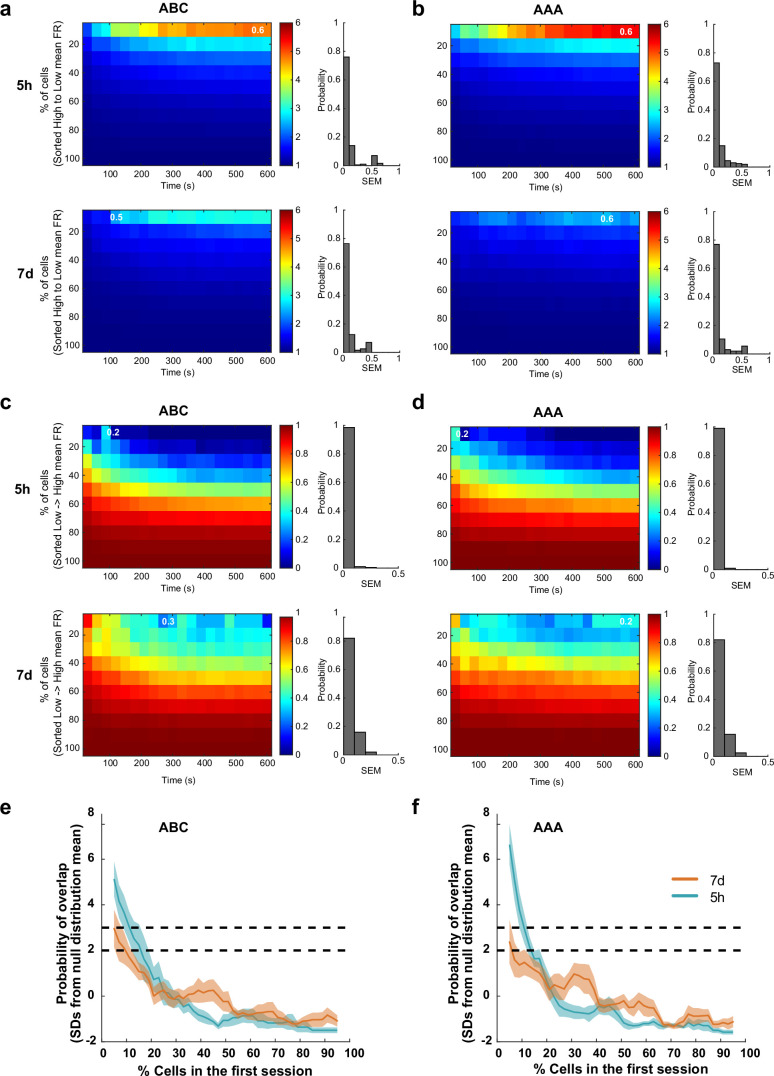
Neuronal activity is correlated with the probability of neuronal overlap. (**a**) The top 10% high-activity cells in context B are likely to be the top 10% high-activity cells in context C 5 h later. Probability of overlap between high-activity cells in context A (7d) or context B (5h) and high-activity cells in context C. Left: Probability of overlap between subsets of cells with different levels of activity during previous context exploration session (A or B) across time in C. Color bars refer to normalized probabilities (chance = 1). Cumulative values were used for x and y-axes (for example, for x-axis, 300 s means 0–300 s; for y-axis, 40 refers to the neurons within the top 40% of high activity). Values represent averages across mice. Right: the distribution of SEM across mice for the figures on the left. Numbers (in the probability of overlap figures) represent the maximum SEM from each plot. (**b**) Similar to figure (**a**) but for AAA condition. (**c**, **d**) Similar to figures (**a**) and (**b**) respectively but the probability of overlap between low-activity cells in contexts experienced 7 d or 5 h before and high-activity cells in the third context is presented under ABC (**c**) and AAA (**d**) conditions. (**e**) Cells were sorted from high to low activity in contexts A or B with a 10% sliding window, 2% steps. Plots show the probability of overlap between subsets of cells (10% ensemble size) from context A (7d) or B (5h) and the top 10% high FR cells from context C. The probability values were z-scored with respect to a null distribution created by randomly subsampling 10% of cells from contexts A or B, 10,000x (that is, results are represented as standard deviation (SD) from the mean of the null distribution). Dashed line: 2 SD and 3 SD thresholds. (**f**) Same comparison as (**e**) but for the AAA condition. (ABC, AAA: n = 9 mice each). Source data

We reasoned that if, similarly to other memory linking paradigms^3,5^, transient increases in intrinsic excitability in the RSC drive neuronal overlap, then the firing rate (FR) of RSC neurons should be similar for contexts explored close in time. Congruently, RSC neurons maintained a similar frequency of calcium transients for contexts explored within 5 h versus 7 days apart (Fig. 1f and Extended Data Fig. 2). We also found that the highly active cells (especially the top 10% of most active neurons) in a context were more likely to be reactivated in a different context 5 h versus 7 days later (Extended Data Fig. 3). Because RSC neurons encode distinct contexts using an FR code^19^, it is likely that neuronal firing dynamics within the RSC also impact the ability to decode context identity. Indeed, a Naive Bayes (NB) classifier performed better at distinguishing sessions recorded 7 days versus 5 h apart (Fig. 1g and Extended Data Fig. 2). Finally, we investigated the coactivity patterns of RSC neurons during these context explorations. Theoretical and experimental models suggest that groups of neurons with synchronized activity encode task-relevant information in the hippocampus, cortical and subcortical regions^20–23^. However, the function of such coactivity patterns during memory formation within the RSC is unclear. Therefore, we calculated the pairwise correlation (PWC) for each pair of RSC neurons within each session (Supplementary Fig. 1). We found that the across-session stability of these PWC maps was higher when contexts are explored on the same day (Fig. 1h,i), indicating that RSC neurons maintain patterns of coactivity when contexts are explored 5 h apart. Together, these data indicate that overlapping RSC ensembles are activated when contextual memories are acquired close in time, and the dynamic activity of these overlapping ensembles may play a critical role in linking different contextual memories.

**Extended Data Fig. 4:**
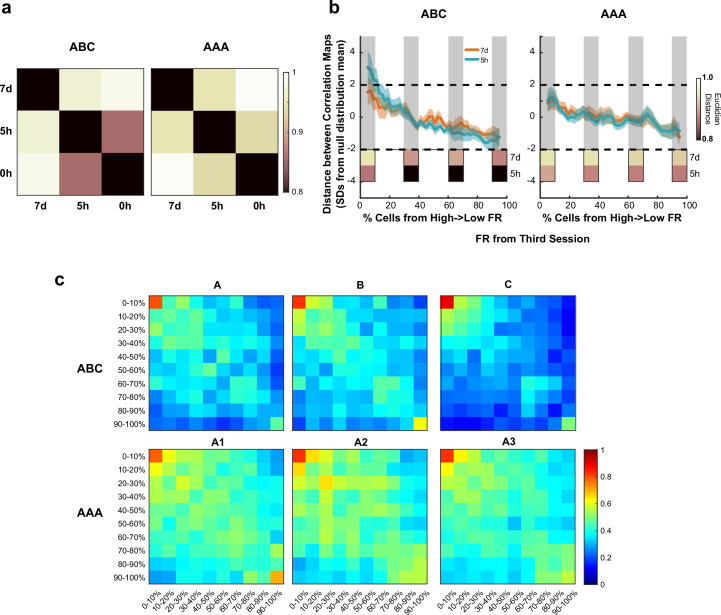
High and low firing rate neurons make differential contributions to representational similarity to regulate memory linking and discrimination. (**a**) Euclidean distance (ED) between correlation maps. For each animal, the ED was calculated for all possible combinations to create an ED map. For each map, all the distances were normalized by the maximum distance. Normalized ED maps were then averaged across animals to produce the plots (ABC, right, AAA, left, n = 9 each). ED between correlation maps in the 5 h interval is lower than for the 7 d interval for the ABC (right) or the AAA (left) contexts conditions. (**b**) Cells were sorted from high to low activity in context C (x-axis) with a 10% sliding window and 2% steps. Correlation maps were calculated by excluding 10% of cells belonging to each of these sliding windows and the ED (y-axis) between contexts explored 7 d or 5 h apart under ABC (right) or AAA (left) condition was calculated. ED values were normalized with to a null distribution created by randomly subsampling 10% of cells from context C, 10,000x (that is, results are represented as standard deviation (SD) from the mean of the null distribution). The 2 SD threshold is labeled with a dashed line. Plots on the bottom of each image show the average ED across animals for the 7 d and 5 h intervals when the following groups of cells are excluded: 0-10%, 30-40%, 60-70%, and 90-100%. Note that the ED for the 5 h interval is always lower than for the 7 d interval. For AAA, the exclusion of any batch of 10% cells does not significantly affect the ED. However, for the 5 h interval in ABC, the top 10% FR cells, when excluded, significantly change the ED. Therefore, the top 10% FR cells are critical for the similarity between correlation maps when different contexts are explored but the contribution of these top 10% FR cells is not significant when the same context is explored at the same time intervals. (**c**) Plots show the average normalized correlation maps across animals during context exploration in ABC (top) or AAA (bottom) conditions. For each animal, cells were sorted from high to low firing rate (based on the last context exploration). The neuronal population was then split into 10% non-overlapping groups. Average Pearson correlation between groups was calculated. A correlation map of the average correlation between groups was constructed and normalized to the maximum average correlation value for each animal. Plots show the average of these normalized correlation maps across all animals. The top FR cells have the highest correlation values for all conditions and sessions. Importantly, for AAA, all correlation maps are similar despite the session. However, for ABC, the maps show larger differences, and the similarity between correlation values between contexts B and C, which are 5 h apart, seem to be higher for the high FR cells, as shown previously. Source data

Although overlap in the underlying neuronal ensembles can link two memories, these memories remain distinct^3,4^. To address how temporally proximate memories can be distinguished while being behaviorally linked, we calculated the functional connectivity difference (Euclidean distance (ED)) between correlation maps of neuronal activity from different sessions of the same animals when different and the same contexts were explored across 7 days versus 5 h apart (Extended Data Fig. 4). We found that excluding 10% of the most active cells from the correlation maps significantly increased the ED between correlation maps when mice explored distinct contexts 5 h apart but not in other imaging conditions (Extended Data Fig. 4). The outsized contribution of high FR cells to representational similarity during the exploration of two distinct contexts (versus the same context) 5 h apart is consistent with their higher probability of reactivation (Extended Data Fig. 3). Overall, these data indicate that high FR cells within the RSC drive overlap and representational similarity between linked memories at an ensemble level, while the representation of the same context is driven more equitably by high and low FR cells. Therefore, memory linking may be driven by highly active cells, while less active cells encode different contextual features relevant to sustaining the independence between contextual experiences^21^.

### RSC neuronal overlap links memories close in time

**Fig. 2:**
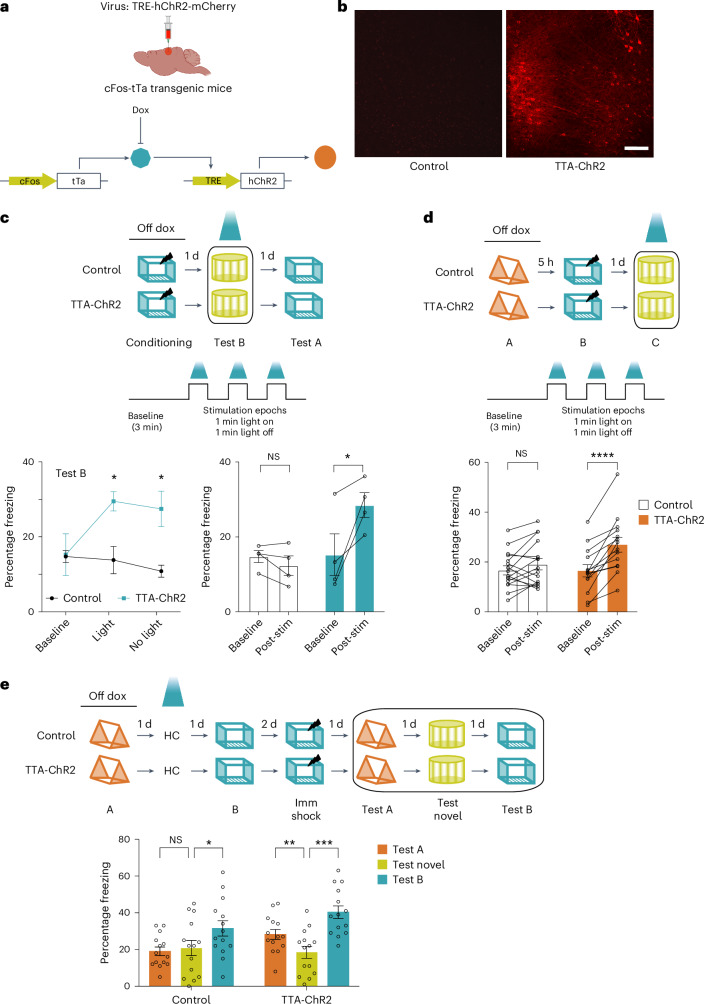
Overlap in RSC neuronal ensembles is sufficient to link contextual memories. **a**, Schematic of the TetTag system: cFos-tTa or wild-type littermate mice were injected with the TRE-hChR2-mCherry virus. **b**, ChR2-mCherry expression in the RSC 1 day after fear learning. Scale bar, 100 µm. **c**, Optogenetic reactivation of an RSC ensemble underlying a fearful context is sufficient for fear expression: top, experimental setup; middle, optogenetic stimulation protocol; bottom, during test B, TTA-ChR2 mice displayed more freezing compared to the control group during the post-baseline stimulation and non-stimulation epochs (*n* = 4 mice per group; two-way repeated-measures (TWRM) analysis of variance (ANOVA), *F*_Interaction_ (2, 12) = 6.95, *P* = 0.009, uncorrected Fisher’s least significant difference). **d**, Optogenetic reactivation of an RSC ensemble underlying a linked memory is sufficient for fear expression: top, experimental setup; bottom, reactivation of the RSC neuronal ensemble tagged during the linked context exploration (context A) increased freezing in cFos-tTa mice during the post-stimulation period, while the freezing in the control group remained unchanged (*n* = 16 and 14 mice for control and cFos-tTa groups; TWRM ANOVA, *F*_Interaction_ (1, 28) = 12.5, *P* = 0.001; Sidak’s test; baseline freezing (control versus TTA-ChR2: *P* = 0.99); post-stim freezing (control versus TTA-ChR2: *P* = 0.046); TTA-ChR2 (baseline versus post-stim freezing: *P* < 0.0001). **e**, Reactivation of the RSC ensemble underlying the first context memory extends the temporal window for memory linking: RSC ensemble tagged during context A was reactivated on the day between the two context exposures separated by 2 days. While control mice did not link the two contexts, reactivation of the first context ensemble led to contextual memory linking in the experimental group: freezing in both previously explored contexts was higher than freezing in a novel context (*n* = 14 mice per group; TWRM ANOVA, *F*_Interaction_ (2, 52) = 3.3, *P* = 0.04; Dunnett’s test; novel versus test B: *P* = 0.046 and 0.0003; novel versus test A: *P* = 0.68 and 0.007 for control and TTA group respectively). Data represent the mean ± s.e.m. and each data point. *P < 0.05, **P < 0.01, ***P < 0.001. Dox, doxycycline; HC, home cage; NS, not significant; Post-stim, post-stimulation; Imm shock, immediate shock. Source data

**Extended Data Fig. 5:**
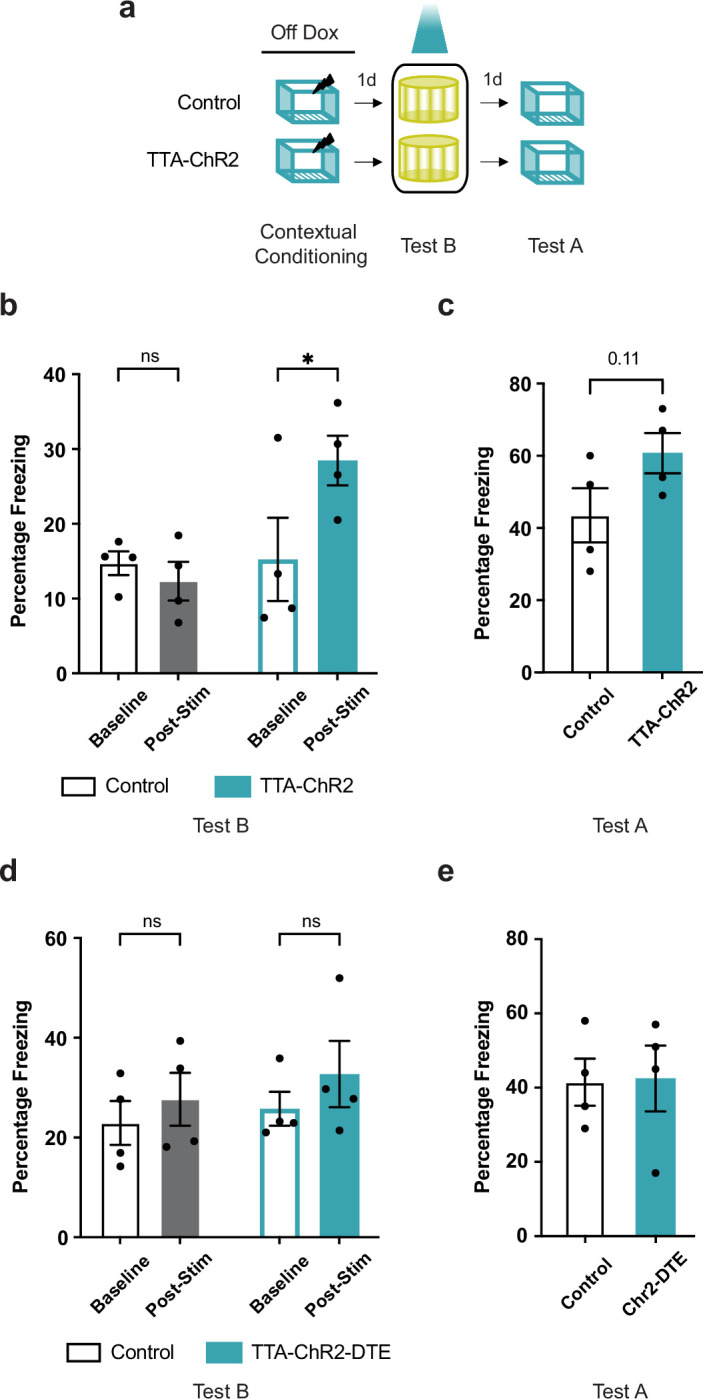
RSC neuronal ensemble (and not dendritic ensemble reactivation) following contextual fear conditioning results in fear expression. (**a**) Experimental setup: cFos-tTa (TTA-ChR2) mice and their wildtype littermates (Control) underwent bilateral viral injections (TRE-ChR2-mCherry) and optic cannula implants. Mice were taken off doxycycline chow (three days before contextual fear conditioning in context A: 2 footshocks, 2 s, 0.7 mA) to allow c-fos promoter-driven tTA and Channelrhodopsin (ChR2) expression. Following contextual fear conditioning, mice were tested in a novel context (Test B) while the previously tagged neurons were activated. The following day mice were retested without any optogenetic manipulation in the training context (Test A). (**b**) During Test B, freezing during the two post-stimulation conditions (with laser and without laser stimulation) was not different. Therefore, freezing during this period is presented together as post-stimulation freezing (TWRM ANOVA, group X time interaction, F (1, 6) = 11.93, *p* = 0.01, Sidak’s post hoc tests, n = 4 each group)). (**c**) During Test A, the TTA-ChR2 mice display comparable freezing to the control mice (t = 1.85, df=6, *p* = 0.11, n = 4 each group). (**d**) Reactivation of previously activated dendrites is not sufficient for fear memory expression: Experimental set up is the same as (**a**), but animals were injected with TRE-hChR2-mCherry-DTE or TRE-mCherry-DTE virus in the RSC to reactivate dendritic segments active during contextual fear conditioning. Both groups display similar freezing during baseline and post-stimulation epochs while testing in a novel context (TWRM ANOVA, group X time interaction, F (1, 6) = 0.26, *p* = 0.6, Sidak’s post hoc tests, n = 4 each group)). (**e**) Both groups (injected with TRE-hChR2-mCherry-DTE or TRE-mCherry-DTE virus in the RSC) display similar freezing during test in Context A (t = 0.09, df=6, *p* = 0.9, n = 4 each group). Data represent mean ± s.e.m. and each data point, * *p* < 0.05, ns = not significant; all comparisons were two-tailed. Source data

To investigate the causal role of RSC neuronal co-allocation in linking contextual memories, we used the TetTag system^24^ to tag and manipulate the RSC neuronal ensembles activated during context exposures (Fig. 2a,b). We found that 4.7% ± 0.42% (relative to 8.5% ± 0.53% cFos-positive) of RSC neurons were labeled following context exposure. Optogenetic reactivation of the RSC ensemble underlying a single contextual fear memory (~6.05% ± 0.53% RSC neurons) induced fear expression in an otherwise neutral and novel context (Fig. 2c and Extended Data Fig. 5a–c)^16^. Notably, fear expression following optogenetic reactivation within the RSC is distinct from similar results within the hippocampus^25^ in that fear expression was sustained throughout the post-stimulation period and not just the ‘light-on’ epochs. These results are consistent with previously published findings^16^ and, from this point onwards, freezing data during optogenetic reactivation are presented as a comparison between the baseline and post-stimulation period. Overall, our data confirm the critical role of RSC and associated brain circuits in processing contextual information^16,17,26–28^.

When two contextual memories are acquired close in time, and one is paired with a fearful stimulus, the mice also consider the second neutral but ‘linked’ context as fearful (that is, the two memories are linked)^3^. We found that optogenetic reactivation of RSC neurons engaged during exploration of the ‘linked’ context after contextual memory linking was sufficient to elicit freezing in mice exploring a novel context (Fig. 2d and Supplementary Fig. 2). We confirmed that such fear expression did not result from the labeling of RSC neurons outside the tagging window (for example, during exposure to context B) or due to differences in contextual learning or linking of contextual memories (Supplementary Fig. 2). First, these data indicate that reactivating the memory of a ‘linked’, but otherwise neutral context, was sufficient to elicit a conditioned response, a result that supports our hypothesis that the recall of one linked memory results in the recall of the other. Second, these findings also demonstrate that manipulation of neuronal ensembles within the RSC alone can drive contextual memory linking.

**Extended Data Fig. 6:**
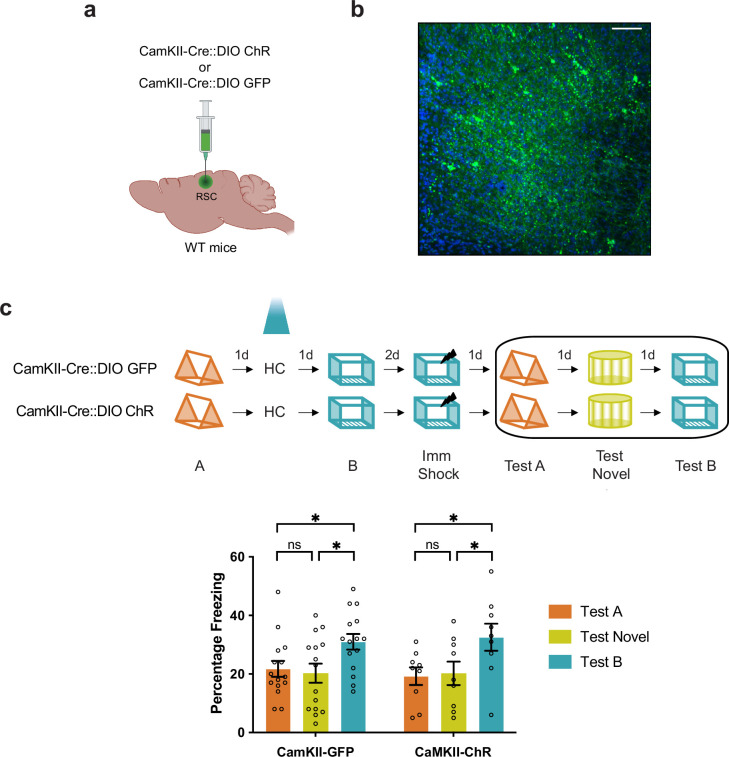
Optogenetic activation of a randomly labeled ensemble does not result in memory linking. (**a**) Mice received a bilateral injection CamKII-Cre::DIO-ChR or CamKII-Cre::DIO-GFP to label a small subset of RSC ensemble. (**b**) Representative image of WT mice injected with CamKII-Cre::DIO-ChR-GFP in the RSC. Scale: 20 µm. (**c**) Control (CamKII-Cre::DIO-GFP, n = 15) as well as experimental (CamKII-Cre::DIO-ChR, n = 9) mice display low levels of freezing in a novel as well as the previously explored neutral (Context A) context but freeze more in the training context (Context B). (TWRM ANOVA, F_time_ (1.9, 42.4) = 9.8, *p* = 0.0004, Tukey’s multiple comparisons test). Data represent mean ± s.e.m. and each data point, * *p* < 0.05. Source data

**Extended Data Fig. 7:**
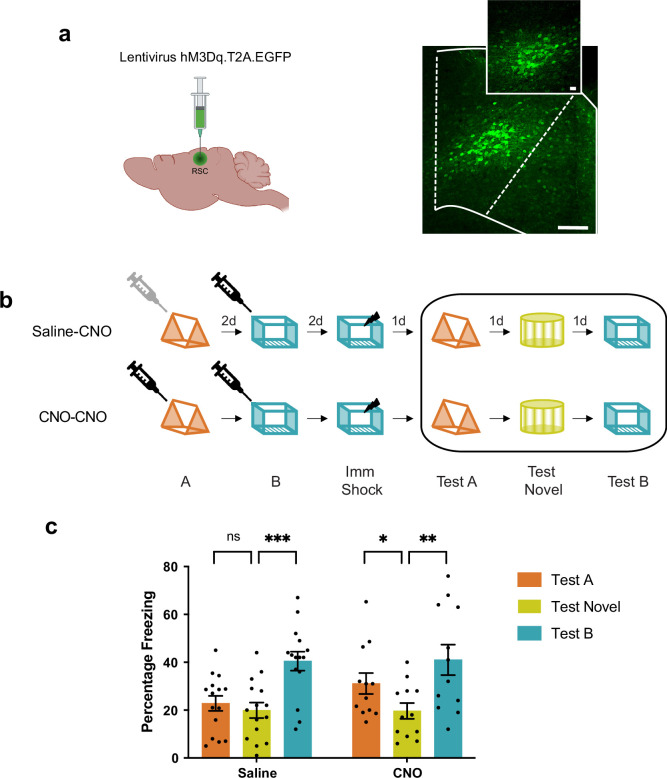
Chemogenetic manipulation of the neuronal ensemble overlap is sufficient to link two distinct contextual memories. (**a**) All mice received a bilateral injection of lentivirus DREADD hM3Dq-T2A-EGFP which infects a sparse population of RSC neurons. Representative images demonstrating hM3Dq-T2A-EGFP infection of RSC neurons from two mice on the left. Scale: 100 and 20 µm. (**b**) All mice explored two different contexts 2 days apart and were subsequently shocked in one of these contexts. Neuronal excitability was increased in a small subset of RSC neurons by administering a CNO (0.5 mg/kg) injection 45 mins before each context exploration. The control mice only received the CNO injection before the second context exploration. (**c**) Control mice display low levels of freezing in a novel as well as the previously explored neutral (Context A) context but freeze more in the training context (Context B). In contrast, mice from the experimental group display memory linking: Both the previously explored contexts (Context A and B) elicit high freezing relative to the freezing in a novel context. (TWRM ANOVA, F_time_ (1.8, 44.9) = 28.45, *p* < 0.0001; Dunnett’s multiple comparisons test; Saline-CNO, n = 15, CNO-CNO, n = 12,). The physical contexts presented were counterbalanced to minimize any effect of context similarity. Data represent mean ± s.e.m. and each data point, * *p* < 0.05, ** *p* < 0.01, *** *p* < 0.001. Source data

While two contexts explored within a day are linked, contexts explored 2 or 7 days apart are not allocated to overlapping neuronal ensembles and, therefore, are not linked^3,29^. We asked if we could link two distant contextual memories (acquired 2 days apart) by artificially biasing a specific RSC neuronal ensemble to encode both memories. We tagged the RSC neuronal ensemble activated during a context exploration (context A) and optogenetically reactivated this ensemble the next day, 1 day before exposure to another context (context B; Fig. 2e). We reasoned that this would reactivate the first memory, maintain the increase in neuronal excitability and, therefore, force the recruitment of this same ensemble^7^ during the exploration of another context a day later. We allowed 24 h for expression and then reactivated the RSC ensemble to allow sufficient expression of Channelrhodopsin after tagging^30^. While two contexts explored 2 days apart are normally not linked, this optogenetic reactivation of the first contextual memory was sufficient to bridge this 2-day gap and drive the linking of two otherwise independent contextual memories (Fig. 2e). We further confirmed the role of neuronal ensemble overlap in the RSC using a chemogenetic system^3^. When we forced the co-allocation of two distinct contextual memories by enhancing the neuronal excitability in the same sparse population of RSC neurons before each context exploration (2 days apart), we found that these memories were linked (Extended Data Fig. 7). Additionally, optogenetic activation of a small but random population of RSC neurons between two context exposures did not link two independent contextual memories (Extended Data Fig. 6). Together, these data demonstrate that neuronal ensemble overlap in the RSC is critical for linking of contextual memories.

### Overlap in dendritic ensembles encoding linked memories

Ours and previous results demonstrate that the allocation of contextual memories to overlapping neuronal ensembles is critical for linking contextual memories^3,29^. However, the intracellular processes that mediate neuronal overlap are poorly understood. Specifically, whether dendritic plasticity mechanisms contribute to neuronal overlap is unclear. Within the overlapping ensembles, linked memories are thought to be encoded by distinct synaptic changes that allow the memories to maintain their distinct identities^31^. There are at least three dendritic hypotheses that could account for memory linking. First, linked memories may be allocated to different dendritic branches within the encoding neurons (dis-allocation). Second, linked memories may be randomly allocated to dendritic branches within the encoding neurons. Third, because experience-dependent dendritic plasticity is highly localized and can affect future plasticity at nearby spines on the same dendritic segment^12,15^, it is likely that following the first context exposure, localized changes in dendritic plasticity temporarily bias the activation of the same dendritic segments during a subsequent context exposure^32^. In this scenario, distinct synaptic changes on the same dendritic branches could drive the co-activation of the same neuronal ensemble and, therefore, linking of two memories. We propose that localized dendritic plasticity is a key mechanism driving neuronal ensemble overlap since such plasticity could affect the propagation of synaptic inputs on specific dendritic segments to the soma.

**Fig. 3:**
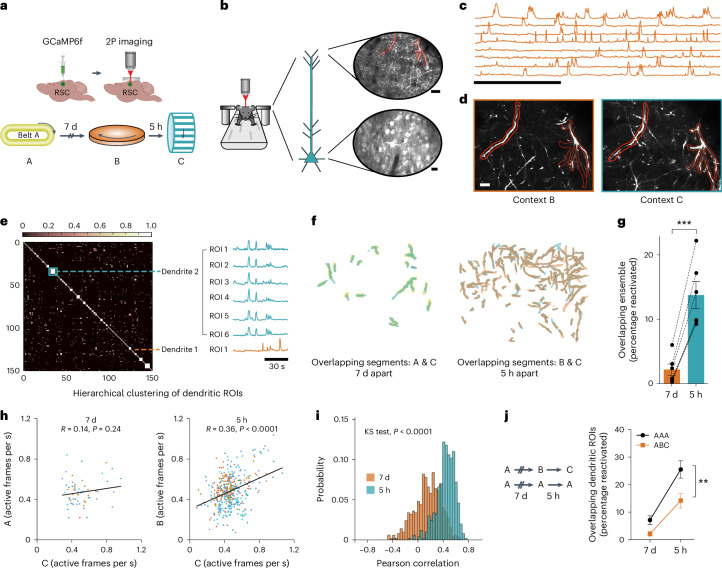
Overlapping dendritic segments encode memories of two contexts explored close in time. **a**, Experimental setup. **b**, RSC neurons and dendritic segments were tracked across 7 days. Maximum intensity projection from one imaging session showing apical dendritic segments (top, scale bar, 20 μm) and layer V RSC neurons (bottom, scale bar, 10 μm). **c**, Representative calcium traces from eight putative RSC dendritic segments. Scale bar, 2 min. **d**, Dendritic segments from **b** tracked across two imaging sessions 5 h apart. Scale bar, 10 μm. **e**, Hierarchical clustering of RSC dendritic ROIs: Sorted cosine similarity matrix of 150 ROI pairs from one animal. Blue box and line depict the correlated calcium activity of 6 ROIs clustered as a single dendrite. Orange line indicates a single ROI that was not clustered with any other ROI. **f**, Example of reactivated (overlapping) dendritic segments from one mouse. **g**, The same dendritic segments are more likely to be activated when context exposures are 5 h apart versus 7 days apart (paired *t*-test; *t* = 9.2; *P* = 0.0003; *n* = 6 mice). **h**, Dendritic activity is more correlated when dendrites are reactivated 5 h (*P* < 0.0001) versus 7 days (*P* = 0.24) apart. Scatterplot of the FRs of all reactivated ROIs in context A (7 days) and context B (5 h) as a function of FRs in context C. Lines represent least-squares linear regression. Data from each mouse are represented in a different color. **i**, To confirm the differences in the number of reactivated dendrites for two context exposures 7 days or 5 h apart did not affect our results in **h**, data from **h** were subsampled (30 ROI pairs, 500×) to generate a probability distribution of Pearson correlations (Kolmogorov–Smirnov (KS) test, *P* < 0.0001). **j**, Dendritic overlap is greater when mice explore the same context versus distinct contexts (TWRM ANOVA, *F*_Context_ (1, 11) = 8.5, *P* = 0.01; Sidak’s test; AAA (5 h versus 7 days), *P* < 0.0001; *n* = 6 and 7 for ABC and AAA groups. Data represent the mean ± s.e.m. and each data point. All comparisons were two tailed. **P < 0.01, ***P < 0.001. Source data

**Fig. 4:**
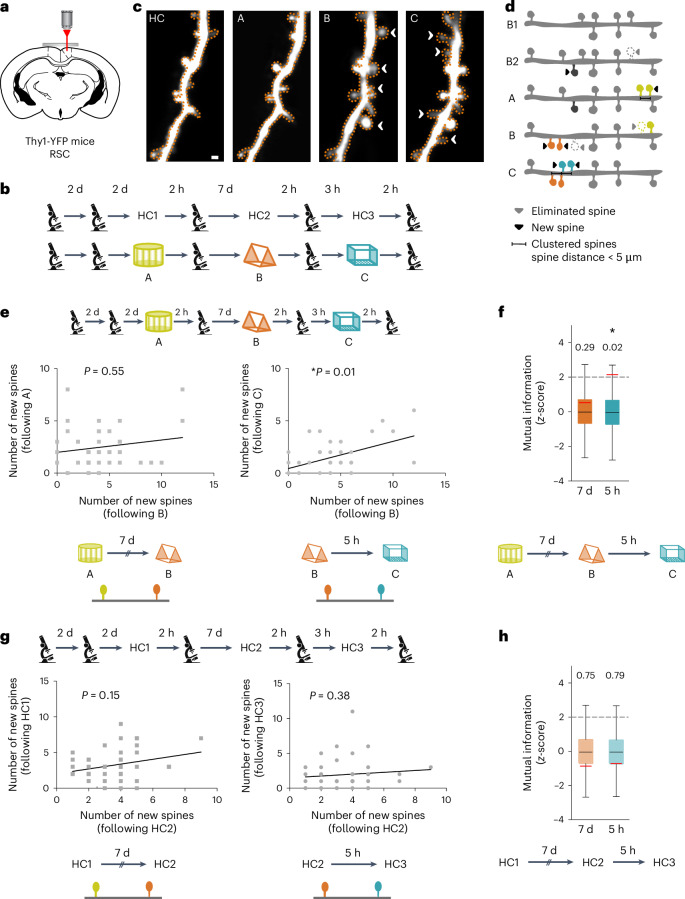
Spines are added to overlapping dendritic segments following memory linking. **a**, Apical RSC dendrites of Thy1-YFP mice were imaged through a cranial window. **b**, Experimental setup. **c**, Representative example of spine dynamics during longitudinal imaging showing clustering of new spines following linked memory formation. Gained spine indicated by a white arrowhead. HC: last baseline imaging session; A, B and C: exposure to contexts A, B and C, respectively. Scale bar, 1 μm. **d**, Schematic of various spine dynamics (spine addition, elimination and clustering) measured. **e**, New spines are likely to be added to the same dendritic segments when contexts are explored close in time. Left: number of new spines added to a dendritic segment following context A and B exposure 7 days apart are not correlated (*ρ* = 0.09, *P* = 0.55). Right: number of new spines added to a dendritic segment following context B and C exposure 5 h apart are correlated (*ρ* = 0.37, *P* = 0.01). Spearman’s correlation (*n* = 45 dendrites, 6 mice); alpha level was adjusted to 0.025 to account for multiple comparisons. **f**, Mutual information between new spines added at 7 days or 5 h apart was higher for spines added following context exposures 5 h versus 7 days apart. Observed values (red line) were compared to the *z*-score of a chance distribution (*n* = 45 dendrites; 6 mice). **g**, For HC controls, the numbers of new spines added to a dendritic segment were not correlated whether imaging sessions are separated by either 7 days (right, *ρ* = 0.22, *P* = 0.15) or 5 h (left, *ρ* = 0.14, *P* = 0.38; *n* = 5 mice). Spearman’s correlation (*n* = 42 dendrites, 5 mice); alpha level was adjusted to 0.025 to account for multiple comparisons. **h**, Mutual information between new spines added at 7 days or 5 h was unchanged in control mice. Observed values (red line) were compared to the *z*-score of a chance distribution (*n* = 42 dendrites, 5 mice). Box plots represent the median as the central mark, 25th and 75th percentiles as box edges and whiskers extend to the most extreme data points; all comparisons were two tailed. Horizontal dashed line in **f** and **h** represents the cutoff for significance at *z*-score = 2. Source data

**Fig. 5:**
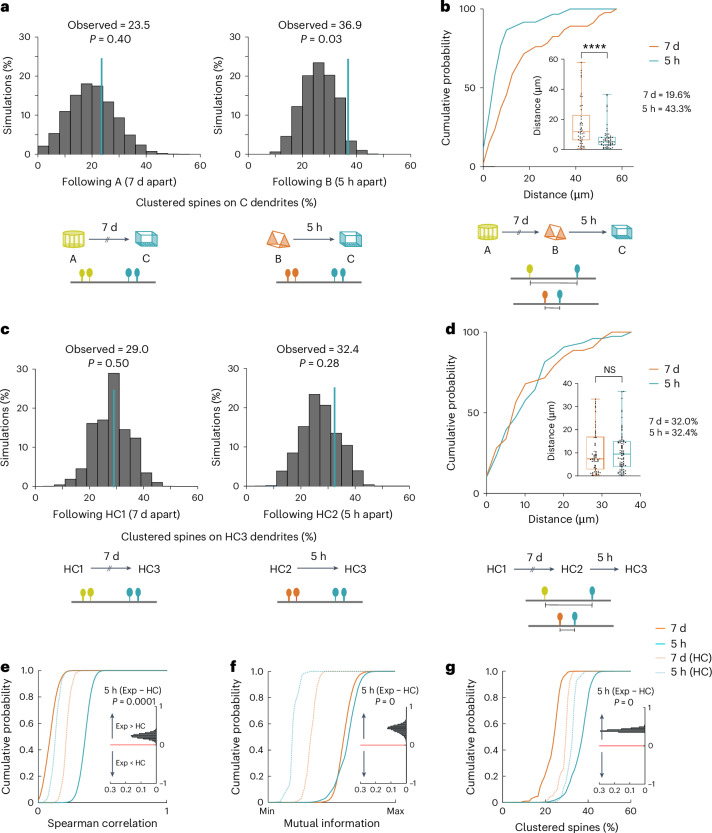
Overlapping dendritic segments gain spine clusters following memory linking. **a**, Clustered spine positions following one imaging session were randomly distributed on the dendritic segment. Percentage of clustered spines co-allocated to the same dendritic segments following contexts explored 7 days (left) and 5 h (right) apart; *n* = 6 mice; 10,000 permutations. **b**, New spines following context exposures 5 h, but not 7 days, apart are formed close to one another (KS test, *P* < 0.0001). Inset: average distance between newly formed spines following context exposures 7 days versus 5 h apart; (*n* = 46 and 60 spine pairs for 7 day and 5 h conditions, 6 mice; Mann–Whitney, *P* < 0.0001). **c**, For HC controls, the percentage of clustered spines that were added to segments also containing clustered spines in a previous imaging session (7 days or 5 h prior) were at chance levels; *n* = 5 mice; 10,000 permutations. **d**, New spines formed in control mice did not co-cluster 5 h or 7 days apart (KS test, *P* = 0.6). Inset: average distance between newly formed spines in home cage controls 7 days or 5 h apart. Mann–Whitney test, *P* = 0.8; *n* = 53 and 76 spine pairs for 7 day and 5 h conditions, *n* = 5 mice. Cumulative frequency distribution and the average distance between nearest neighboring spines are different between the experimental and HC groups for imaging sessions performed 5 h apart (KS test, *P* < 0.0015 and Mann–Whitney, *P* = 0.02, respectively). **e**–**g**, Forty dendritic branches for each condition were randomly subsampled (10,000×) to calculate a cumulative distribution of Spearman’s rho (*ρ*) (**e**), mutual information (**f**) and percentage of clustered spines (**g**). Insets demonstrate that Spearman’s rho (*ρ*) (**e**), mutual information (**f**) and the probability of gaining a clustered spine on a segment (**g**) already containing a clustered spine during a previous session (all *P* values < 0.0001), is higher for resampled experimental versus HC group at the 5 h interval. Data points were resampled from the same distributions and hence are not independent of one another. In the box plots, the central mark represents the median, box edges indicate the 25th and 75th percentiles, whiskers indicate the most extreme data points, each data point. All comparisons were two tailed. Exp, experimental. Source data

**Extended Data Fig. 8:**
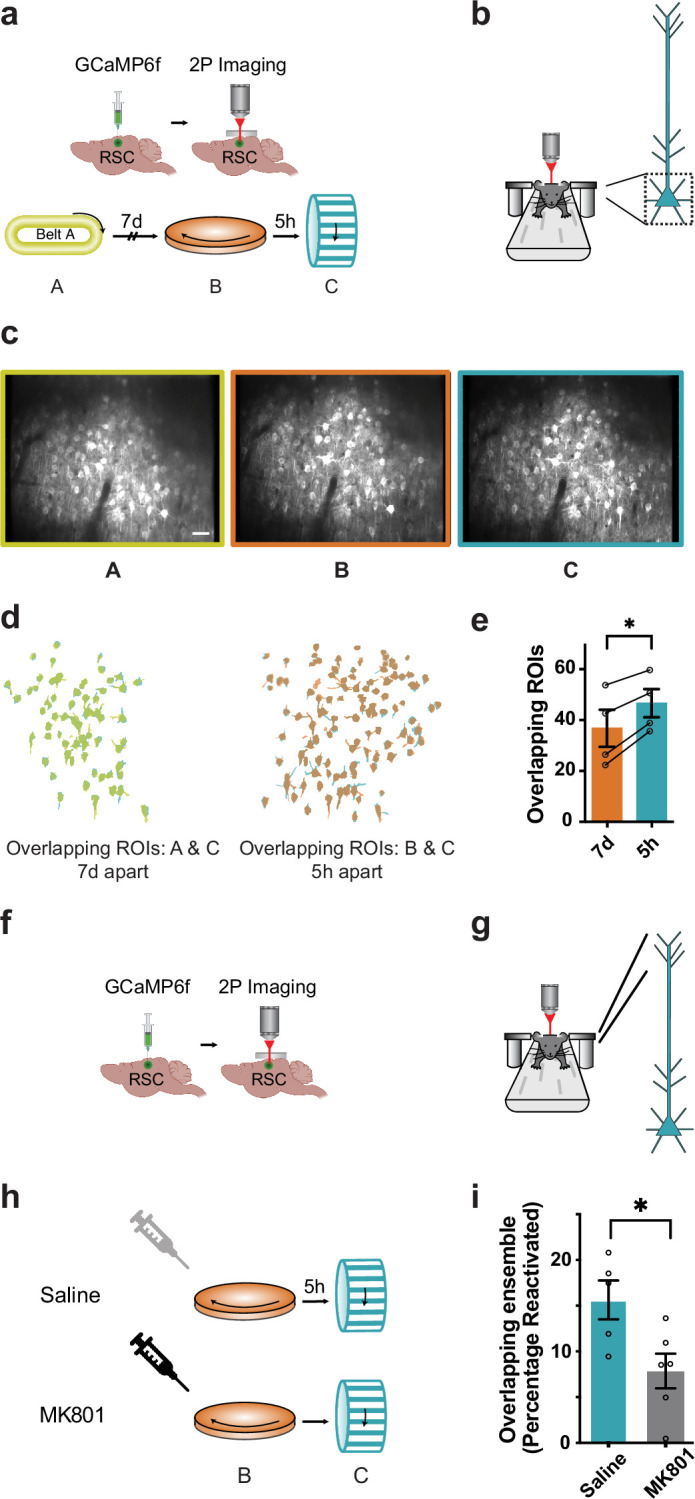
Reactivation of dendritic ensembles is accompanied by overlap in neuronal ensemble in head-fixed mice and requires NMDA receptor activation. (**a**, **b**) Experimental setup: Head-fixed mice experienced three distinct contexts either 7 days or 5 hours apart while calcium transients from layer V RSC neurons were imaged. (**c**) Mean frames from three imaging sessions from a mouse. Scale: 40 µm. (**d**) Overlapping neuronal ROIs reactivated when contexts are separated by 7 days (left) or 5 hours (right) from one mouse. (**e**) The same neuronal ensemble is more likely to be activated in a head-fixed setting when context exposures are 5 hours (5 h) apart vs. 7 days (7 d) apart. (paired t-test; t = 5.6; *p* = 0.01; n = 4 mice). (**f**-**h**) NMDA receptor activation is required for the reactivation of dendritic ensembles. Dendritic overlap was measured as described in Fig. 3. Mice were administered NMDA receptor antagonist, MK801, 30 minutes prior to the first context exposure. (**i**) NMDA receptor antagonist, MK801, impairs reactivation of dendritic ensembles following two context exposures 5 hours (5 h) apart (unpaired t-test; t = 2.7; *p* = 0.02; n = 5 and 6 mice in control and MK-801 group each). Data represent mean ± s.e.m. and each data point; all comparisons were two-tailed. Source data

To distinguish between these different hypotheses, we used two-photon microscopy to investigate the functional and structural dynamics of the apical dendrites of layer V RSC neurons (Figs. 3–5). Specifically, we targeted the apical dendrites of layer V RSC neurons because we have previously demonstrated that plasticity within these dendritic compartments facilitates the formation of single contextual memories^33^ making them an excellent candidate for co-allocation of dendritic plasticity following memory linking. First, we performed longitudinal calcium imaging of the somas and apical dendrites of layer V RSC neurons (see Methods, Supplementary Video 1 and Fig. 3a–d) while mice explored distinct contexts in a head-fixed setting (Supplementary Fig. 3). Like our results in freely moving mice (Fig. 1), head-fixed mice also represent two contexts experienced close in time by recruiting overlapping RSC ensembles (Extended Data Fig. 8). We next assessed the degree of overlap among RSC apical dendritic branches (layer I, ~30 μm from pia mater) when contexts are explored close in time. The same dendritic regions of interest (ROIs) were preferentially reactivated as mice explored two distinct contexts 5 h, but not 7 days, apart (Fig. 3e–g,j). Consistent with the role of NMDA receptor activation in memory linking^3^ and clustered spine formation in the RSC^33^, reactivation of dendritic segments required NMDA receptor activation (Extended Data Fig. 8).

The extent and prevalence of independent dendritic and somatic events during calcium imaging, the causality or direction of their interdependence and the factors that affect these are poorly established^34–37^. For our experiments, we sought to minimize the effect of backpropagating action potentials and global dendritic transients by imaging RSC apical tuft dendrites, as apical tuft branches display a degree of independence from one another and somatic calcium events^37^. To account for highly correlated calcium transients across ROIs, we performed a hierarchical clustering analysis to group segmented ROIs into single dendritic units when their calcium dynamics are highly correlated (Fig. 3e). We found similar proportions of clustered ROIs among reactivated and overall segmented ROI populations (reactivated ROIs: 0.85 ± 0.02; total ROIs: 0.86 ± 0.02; *P* = 0.3). Clustering segmented ROIs (to account for global dendritic transients or backpropagating action potentials contaminating our results) did not change our observed effects (Fig. 3f,g and Supplementary Fig. 4). It is still possible that there is a one-to-one correspondence between our reactivated neurons and dendritic segments (all clustered ROIs), but we believe this is unlikely given the low levels of clustering (1.15 ± 0.03 ROIs per cluster; Supplementary Fig. 4b) and the large difference between neuronal and dendritic overlap in our head-fixed experiments (Fig. 3g and Extended Data Fig. 8). Differences in neuronal and dendritic overlap are unlikely to be due to lower signal-to-noise ratio during dendritic imaging: Neuronal overlap using one-photon and two-photon imaging (Fig. 1 and Extended Data Fig. 8) is similar despite different signal-to-noise ratios and different than the degree of dendritic overlap. Importantly, these results do not rely on using any particular clustering criteria, as clustering cutoffs that consistently resulted in clustered ROIs within shuffled distributions (randomized activity) yielded low cluster sizes in the experimental dataset (clustering cutoff = 0.3, 1.39 ± 0.06 ROIs per cluster; see Methods) and similar overlap results (*P* < 0.001).

Next, we analyzed calcium transient frequencies within the reactivated dendrites during two context exposures and found that these were highly correlated when contexts were explored 5 h but not 7 days apart (Fig. 3h,i). These data indicate that the synaptic drive and the local excitability mechanisms driving dendritic activity are maintained during the encoding of linked memories. Finally, we found that dendritic ROIs were more likely to be reactivated closer in time (5 h versus 7 days) whether animals experienced the same or different contexts (Fig. 3j). In addition, we confirmed that dendritic representations are more similar for the same context versus different contexts irrespective of time. Overall, the data presented here (Fig. 3) are consistent with the hypothesis that local dendritic mechanisms govern the allocation of two contextual memories encoded close in time to the same dendritic segments.

### Spine remodeling in overlapping dendritic segments

**Extended Data Fig. 9:**
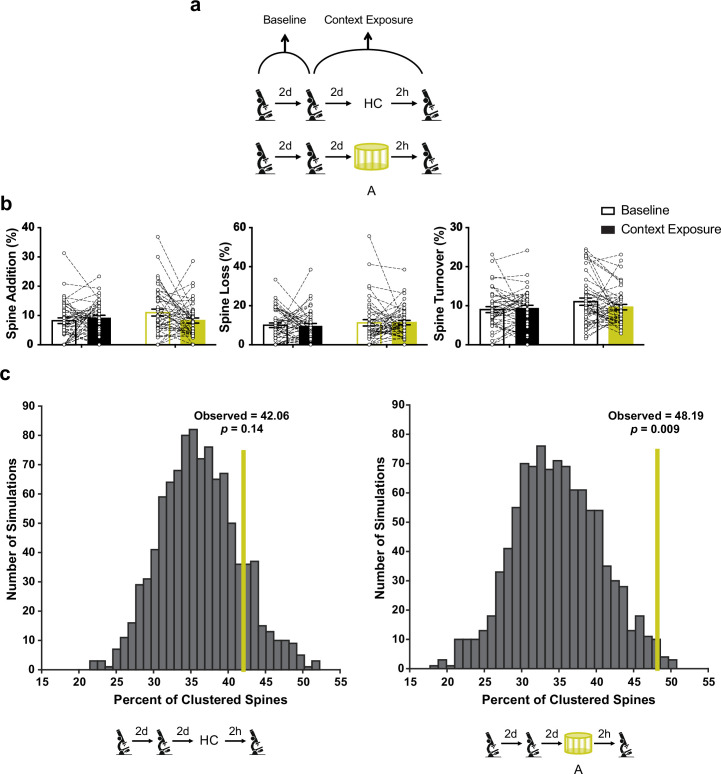
Spine dynamics within the RSC following context exposure. (**a**) Thy1-YFP mice were imaged every two days (baseline), and the same RSC dendrites were tracked to measure contextual exposure-related spine dynamics. Following two baseline imaging sessions, mice were left in the home cage or exposed to a novel context. (**b**) Spine addition, spine loss, and spine turnover is not altered within the RSC apical dendrites following context exposure (TWRM ANOVA; Sidak’s post hoc tests;). Control: n = 44 dendrites (5 mice); Experimental: n = 46 dendrites (6 mice). (**c**) Clustered spine addition following context exposure is greater than chance: The histogram shows percent clustering from 1000 simulations of randomized new spine positions, where the percent of new spines within 5 µm of each other was calculated. Yellow line: Percentage spine clustering observed from the data. The percentage of clustered spines is more than that expected by chance for the experimental group (Right, n = 6; *p* = 0.009) whereas the percentage of clustered spines is at chance levels for the control group (Left, n = 5; *p* = 0.14). Control: n = 44 dendrites (5 mice); Experimental: n = 46 dendrites (6 mice). Data represent mean ± s.e.m. Source data

Given that overlapping dendritic segments are activated when contexts are experienced close in time, we next investigated whether learning-related spine dynamics were also evident on the same dendritic segments. Within the RSC, contextual memory formation is accompanied by structural plasticity at apical dendritic branches of layer V neurons, such that spine addition is clustered on small stretches (~5 μm) of a dendritic segment^33^. These data are consistent with the clustered plasticity hypothesis and indicate that experience-dependent spine remodeling is spatially restricted in a branch-specific manner^11,12,33^. We used in vivo two-photon microscopy to image spines on the RSC apical dendrites of Thy1-YFP-H mice following multiple context exposures (Fig. 4a–d). We confirmed that mice still display memory linking under these conditions (Supplementary Fig. 5). We found that relative to spine dynamics during a baseline period, novel context exposure did not change overall spine addition, spine loss or spine turnover (Extended Data Fig. 9). However, following context exposure (but not in control mice) new spines were added in clusters (or within 5 μm of each other; Extended Data Fig. 9). Hence, consistent with previous findings^33^, novel context exploration results in clustered plasticity on RSC dendrites.

Since our results showed reactivation of the same dendrites during context exposures close in time, we next investigated the possibility that spines added following these context exposures also tend to be added to the same dendritic segments (Fig. 4e–h). We found a positive correlation between the number of spines added to the same dendritic segments following two context exposures 5 h apart (Fig. 4e). Our observed correlation coefficient was unlikely to be observed by chance (new spines added to a dendritic segment following one imaging session were shuffled to generate a randomized set of correlation values; 10,000×; *P* = 0.006). In contrast, the number of spines added to a dendritic segment when two distinct contexts were explored 7 days apart as well as in home cage conditions were not correlated (Fig. 4e,g) and not different from correlation coefficients observed following the shuffling procedure (none of the *P* values were statistically significant). Furthermore, the number of spines lost was not correlated under any imaging interval in either group (Supplementary Fig. 6).

We also calculated the mutual information contained in the number of spines added following two context explorations 5 h apart and found that spine addition following the encoding of one context is predictive of the number of spines added following the encoding of a linked context. This was not true for other imaging conditions (Fig. 4f,h). Finally, the correlation coefficient generated by distributions of newly added spines following two context exposures 5 h apart was statistically different than the correlation values generated in other conditions (Fisher transformation; 5 h, experimental versus HC; experimental, 5 h versus 7 days; and HC, 5 h versus 7 days; *Z* = 1.8, 1.67 and −0.42; *P* = 0.03, 0.047 and 0.33, respectively). To control for the differences in the number of dendritic branches imaged under different conditions, we subsampled 40 dendritic branches from each condition (10,000×) to obtain a distribution of Spearman correlations and mutual information. We found that Spearman correlation and mutual information values were higher when mice explored two novel contexts 5 h apart compared to all other conditions (Fig. 5e,f and Supplementary Fig. 7). These data indicate that spine addition is biased to the same RSC dendritic segments when contextual memories are linked (acquired 5 h apart), but not when these memories are independent (acquired 7 days apart).

Next, we looked for co-allocation of clustered spines by asking whether dendritic segments that gained clustered spines during a context exposure were also the ones that gained clustered spines during a previous context exposure. We found that the spine clusters were more likely to be added to the same dendritic segments when contexts were explored 5 h apart (Fig. 5a). In contrast, the addition of clustered spines in the control group, and following context exposures 7 days apart, was at chance levels (Fig. 5a,c). Using a similar analysis as that used for Fig. 5e,f, we found that the probability that clustered spines were added to a dendritic branch already containing clustered spines was higher for an imaging session when two contexts were explored 5 h apart in comparison to an imaging session at the same time interval in control mice (Fig. 5g). Thus, synaptic plasticity in the form of clustered spine addition following the encoding of linked memories is biased to overlapping dendritic segments.

Finally, we asked whether new spines added following exposure to two linked contexts were added close to one another, or if they cluster with each other. While 43.3% of newly formed spines following the last context exposure were clustered with the spines added following the context exposure 5 h before (average distance between nearest neighbors, Dis_NN_ = 7.7 μm ± 1.0 μm), co-clustering was observed in 19.6% of the new spines when context exposures were 7 days apart (Dis_NN_ = 17 μm ± 2.3 μm; Fig. 5b,d). In control mice, spine co-clustering was similar for 5 h or 7 day intervals (32.4% and 32.0% co-clustered spines; Dis_NN_ = 10.7 μm ± 1 μm and 10.7 μm ± 1.3 μm for 5 h and 7 days, respectively). Synaptic clustering can likely facilitate nonlinear summation of dendritic inputs, which would result in more robust propagation of inputs to the soma, resulting in increased somatic firing^8,38–41^. Indeed, clustered spines are more effective at influencing neuronal spiking and thus the tuning properties of a neuron^42–44^. We demonstrate that new spines and spine clusters are added to overlapping dendritic segments following the formation of linked memories, and these newly formed spines cluster with each other. Such clustered plasticity could facilitate future ensemble activation. Together, the structural and functional imaging data from RSC dendrites indicate that the same dendritic branches are recruited to encode contextual memories formed close in time.

### Reactivation of tagged dendritic ensemble can link memories

**Fig. 6:**
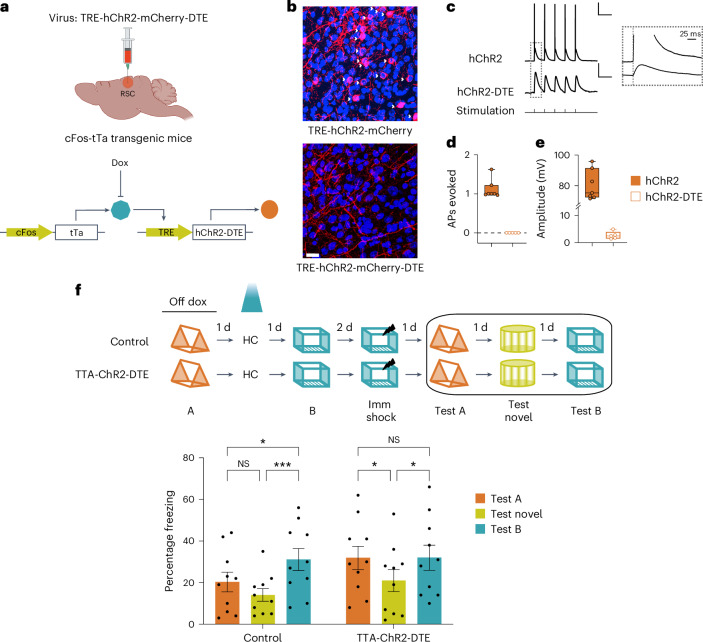
Optogenetic reactivation of RSC dendritic ensembles links contextual memories. **a**, TRE-hChR2-mCherry-DTE virus was injected into cFos-tTa mice to express Channelrhodopsin in the recently activated dendritic segments of cFos-expressing neurons. **b**, Representative RSC images of cFos-tTa mice injected with TRE-hChR2-mCherry-DTE and TRE-hChR2-mCherry showing selective expression of Channelrhodopsin in dendritic segments in the presence of DTE. White arrowheads indicate somatic expression of hChR2. Scale bar, 20 µm. **c**, Whole-cell patch-clamp recordings from RSC neurons of cFos-tTa mice tagged using TRE-ChR2 or TRE-ChR2-DTE constructs. Representative waveforms showing optogenetic stimulation of RSC neurons from TTA-ChR2 and TTA-ChR2-DTE mice resulted in action potentials and transient depolarizations, respectively. Scale bars, 20 mV (top), 1 mV (bottom); 250 ms. Inset shows a magnified view of the first optogenetic stimulation showing response latencies of the stimulus onset. Scale bar, 25 ms. **d**,**e**, Average number of action potentials (APs) elicited (**d**) and response amplitudes (**e**) in TTA-ChR2 and TTA-ChR2-DTE mice. (Mann–Whitney test, *P* = 0.0025; TTA-ChR2: *n* = 7 cells (3 mice) and TTA-ChR2-DTE: *n* = 5 cells (3 mice) for **d** and **e**). **f**, Experimental setup: top, mice explored two contexts 2 days apart. On the day between the two context exposures, the dendrites activated during the first context exposure were reactivated. Bottom: reactivation of context A dendrites, on the day between exposures to contexts A and B, resulted in high freezing in both the previously explored contexts (context A: linked context and context B: shock context) relative to freezing in a novel context. The control mice froze similarly in context A and a novel context, but the freezing in context B (shock context) was higher than freezing in context A or a novel context (*n* = 10 mice each for control and cFos-tTa groups; TWRM ANOVA, *F*_time_ (2, 36) = 14.11, *P* < 0.0001; Dunnett’s multiple-comparisons test). Box plots represent the median as the central mark, 25th and 75th percentiles as box edges, the whiskers extend to the most extreme data points, each data point is presented. Column graphs represent the mean ± s.e.m. and each data point. All comparisons were two tailed; *P < 0.05, ***P < 0.001. Source data

**Extended Data Fig. 10:**
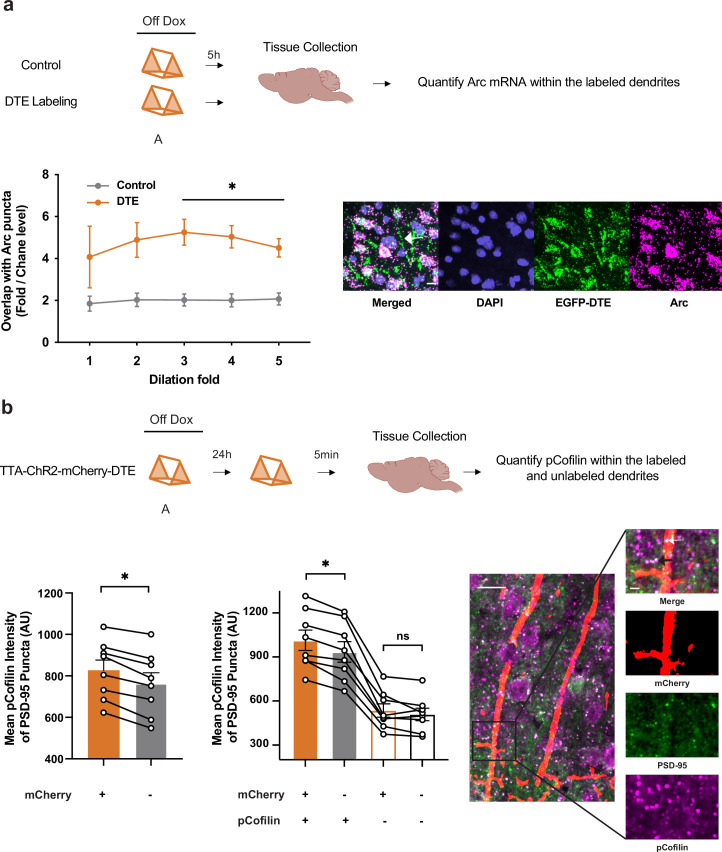
DTE-mediated targeting labels recently activated dendritic segments. (**a**) Dendritic segments labeled using DTE-mediated strategy are enriched in *Arc* mRNA. Top: Experimental Design: Control group was designed to label a small but random subset of dendrites (CamKII-Cre::DIO-GFP in WT mice), and the DTE group used a low titer injection to label activated dendrites sparsely (cFos-tTa mice, TRE-Opsin-GFP-DTE). Bottom: Regions of interest (ROI) were manually delineated to specifically isolate the fluorescent signal within dendrites (to exclude somatic regions). GFP and *Arc* signals within these ROIs were automatically segmented. A 1-5 fold dilation of the GFP signal was applied, and the volume of overlap between the dilated GFP signal and the *Arc* signal was quantified to determine the extent of their colocalization. *Arc* mRNA was enriched in labeled dendrites in the DTE vs. Control group (Control: n = 3, DTE: n = 5; TWRM ANOVA, F_Group_ (1, 6) = 10.08, *p* = 0.02, Sidak’s multiple comparisons test). Scale: 10 µm. (**b**) Dendritic segments labeled using DTE-mediated strategy are preferentially reactivated upon reexposure to the original labeling context. Top: Experimental Design. Bottom Left: PSD-95 puncta on DTE labeled dendrites displayed more pCofilin labeling (n = 8 slices, 4 mice); Wilcoxon test, *p* = 0.008. Bottom Middle: Similarly, PSD-95 puncta that were classified as positively labeled for pCofilin (pCofilin+ PSD-95 + ) displayed higher fluorescence intensity when present on mCherry-labeled dendrites than neighboring pCofilin+ PSD-95+ puncta (TWRM ANOVA, F_Group_ (3, 21) = 137.7, p < 0.0001, Sidak’s multiple comparisons test). Bottom right: Representative image depicting pCofilin-positive puncta (magenta) on PSD-95 (green) and mCherry-positive dendrite (red). White and black arrows represent pCofilin-positive, PSD-95-postive puncta on mCherry-positive dendrites and neighboring regions respectively. Scale: 10 µm, Inset Scale: 2 µm. Data represent mean± s.e.m. and each data point, * *p* < 0.05. Source data

Next, we tested whether such dendritic co-allocation is sufficient for linking contextual memories. We combined the activity-dependent labeling of the cFos-tTA system with the dendritic targeting element (DTE) of *Arc* mRNA, which is selectively targeted and locally translated in activated dendritic segments following learning^45,46^ (Fig. 6a,b). This new approach allowed us to manipulate dendritic activity by expressing Channelrhodopsin in recently activated dendritic segments^47,48^ of RSC neurons that underlie the contextual memory trace. To confirm that we can target recently activated dendrites, we first verified that following DTE-based labeling, mRNA encoding the fluorescent tag and *Arc* colocalize near each other in dendritic compartments (Extended Data Fig. 10). We also confirmed that labeled dendritic segments are more likely to be reactivated upon reexposure to the original tagging stimuli (exposure to the original context). Synaptic activity results in rapid phosphorylation (2–7 min) of Cofilin protein in the synapse^49^, and we found that PSD-95 puncta on labeled dendritic segments displayed an increase in phosphorylated Cofilin protein (Extended Data Fig. 10b). These data support our hypothesis that the DTE-based labeling allowed us to tag dendrites in an activity-dependent manner.

We assessed whether activation of dendritic segments tagged in this manner results in somatic activation. While optogenetic stimulation of RSC neurons from TTA-ChR2 mice resulted in action potentials, the same stimulation only elicited transient small-amplitude depolarizations in the TTA-ChR2-DTE mice (Fig. 6c–e). Moreover, we also tagged and reactivated RSC dendrites activated during contextual fear conditioning in a novel context (Extended Data Fig. 5d,e). Unlike our somatic manipulations, tagging and activation of dendritic compartments alone were not sufficient to elicit fear expression. Together, these data indicate that Channelrhodopsin-mediated dendritic activation using the TTA-ChR2-DTE system has limited effects on the depolarization of somas and, therefore, fails to elicit an acute behavioral response. Hence, combining the cFos-tTA system with DTE allowed us to study the role of previously active dendrites (while limiting somatic involvement) in memory linking.

Next, we asked whether artificially biasing dendritic allocation, like neuronal manipulations (Fig. 2e), is sufficient to link two contextual memories, which would otherwise be independent. We tagged active dendrites during the first context exploration (context A), reactivated these dendrites the next day, and 1 day after this reactivation (or 2 days after context A exploration), we exposed the mice to another novel context (context B). Like the reactivation of context A neurons (Fig. 2e), the reactivation of dendrites first activated in context A was sufficient to link the two contexts (Fig. 6f). Hence, the reactivation of dendrites tagged during the exploration of one context is sufficient to link that context to another independent context. Our results demonstrate a causal role for RSC dendritic mechanisms in the allocation and linking of contextual memories and reveal a new set of rules that govern how linked and independent memories are allocated to various dendritic compartments.

### Biophysical modeling of dendritic plasticity mechanisms

**Fig. 7:**
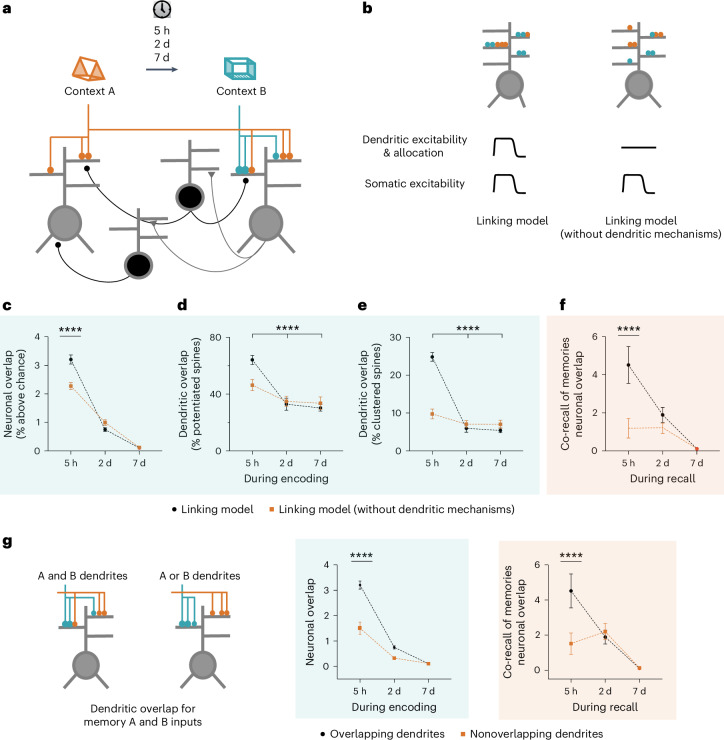
Dendritic mechanisms are necessary for linking memories acquired close in time in a spiking network model. **a**, Spiking network model: network consists of two-layer excitatory neurons (gray) with dendritic subunits, and subpopulations of dendrite-targeting and soma-targeting interneurons (black). **b**, Details of the learning-related plasticity mechanisms within the two network models: memory formation results in increases in somatic and dendritic excitability, and synapses are more likely to be potentiated in the presence of preexisting potentiated synapses on the same dendrite. Learning-related changes in dendritic excitability and probability of synaptic potentiation are eliminated in the linking model without dendritic mechanisms. **c**–**e**, Neuronal overlap (**c**), overlap between dendritic branches containing potentiated synapses (**d**) and overlap between dendritic branches containing newly added clustered spines (**e**) for encoding of two memories acquired 5 h, 2 days or 7 days apart. When dendritic mechanisms are removed from the model, overlap between these measures is reduced when memories are acquired 5 h apart. **f**, Co-recall of two memories as measured by neuronal overlap during recall. Without dendritic mechanisms, neuronal overlap during recall is similar whether memories are acquired 5 h, 2 days or 7 days apart, indicating a lack of memory linking. TWRM ANOVA, *F*_Interaction_ (2,36) = 17.8 (**c**), 54.7 (**d**), 344.4 (**e**) and 61.9 (**f**); all *P* values < 0.0001, Dunnett’s post hoc test. For simplicity, only comparisons within the linking model without dendritic mechanisms are presented. **g**, Dendritic overlap allows somatic overlap and co-recall of memories. Inputs representing context A and B impinge on overlapping or separate dendrites (dendritic overlap is eliminated). During encoding and recall, the neuronal overlap was reduced between groups at 5 h but not 2 day and 7 day time intervals (Sidak’s post hoc test, *P* < 0.0001). When memories are encoded by nonoverlapping dendrites, neuronal overlap is similar between 5 h, 2 day and 7 day groups (TWRM ANOVA, encoding: *F*_Interaction_ (2, 36) = 25.49, recall: *F*_Interaction_ (2, 36) = 66.2; all *P* values < 0.0001, Dunnett’s post hoc test). Neuronal overlap represents the percentage above chance overlap. Data represent the mean ± s.e.m. of ten simulation trials. ****P < 0.0001. Source data

Our data thus far suggest that synergism between somatic and dendritic mechanisms sculpts memory allocation within the RSC to regulate the linking of memories. To explore whether linking of memories acquired close in time is even possible in the absence of dendritic mechanisms, we adapted a network model of memory allocation^32,33^ and used it to investigate how two independent memories can first become linked in a brain region. The model incorporates somatic and dendritic allocation mechanisms that rely on the modulation of intrinsic excitability^10,14,50^ (Fig. 7a,b). As with our experimental data, the network model shows higher neuronal and dendritic overlap as well as increased synapse clustering (Fig. 7c–e) when memories are acquired close in time. Importantly, our model predicts that when linked memories (or memories acquired 5 h apart) are recalled, they maintain higher neuronal overlap indicating co-recall and thus stable linking of these memories (Fig. 7f). To dissect the relative contributions of somatic versus dendritic mechanisms in memory linking, we asked how these neuronal and dendritic overlap measures change in the absence of dendritic allocation and plasticity mechanisms (see Methods). Remarkably, both neuronal and dendritic overlap during encoding is reduced when the model lacks dendritic mechanisms (Fig. 7c–e). More importantly, the lack of dendritic mechanisms in the model abolished co-recall or linking of memories, suggesting that dendritic mechanisms are crucial for stable memory linking (Fig. 7f).

To assess the importance of converging synaptic input onto the same dendritic compartments for memory linking, we modeled synaptic inputs representing the two contexts on separate (exclusively nonoverlapping) dendritic branches. The model predicts impaired neuronal overlap (during encoding and recall) when two memories encoded 5 h apart recruit nonoverlapping dendritic populations, suggesting that to effectively link separate inputs within a neuron, these inputs need to overlap onto the same dendritic compartments (Fig. 7g). Together, our data indicate that dendritic allocation mechanisms may be necessary (Fig. 7) and sufficient (Fig. 6) for linking memories acquired close in time.

## Discussion

Our findings demonstrate that localized dendritic mechanisms play a critical role in mediating neuronal ensemble overlap and thus linking of contextual memories. We demonstrate that, in addition to neuronal ensemble overlap, local dendritic rules further sculpt the allocation of memories to dendritic segments, such that temporally proximate (or linked) memories are likely to be allocated to the same dendritic segments, while temporally distant (or independent) memories are not. We leveraged activity-dependent targeting of dendritic segments to demonstrate that biasing memory allocation to the same dendritic segments is sufficient to link these memories. Accordingly, computational modeling supports the key role of dendritic mechanisms in memory linking. Altogether, the findings presented here demonstrate that localized dendritic mechanisms are critical for linking memories.

### RSC neuronal mechanisms underlie memory organization

Within the RSC, neuronal ensemble activation encodes contextual and spatial information^16,19,37^, and plasticity within the RSC is necessary for contextual fear expression^28^. We discovered that RSC representations of two contextual memories acquired close in time, such that they are behaviorally linked, are more similar than those of two memories acquired a week apart (independent memories; Fig. 1). Consistent with this, we show that optogenetic reactivation of the RSC ensemble underlying a linked neutral memory is sufficient to induce fear expression associated with a second fearful memory (Fig. 2d). We also demonstrate that optogenetic or chemogenetic manipulation of RSC ensemble overlap alone is sufficient to link two otherwise independent memories (Fig. 2e and Extended Data Fig. 7). Together, our data demonstrate that RSC ensemble overlap alone is sufficient to link distinct contextual memories.

We find that neuronal overlap in the RSC, like overlap in the CA1 ensembles, can affect the linking of contextual memories. Activity within the hippocampus and the RSC is important for contextual and spatial tasks^16,25^. While the precise role and the interaction of hippocampal and retrosplenial subregions in contextual memory processing are not well understood, optogenetic reactivation of tagged RSC ensembles can result in fear expression even when hippocampal activity is inhibited^16^. These data and the RSC’s well-established projections to the important nodes in the fear circuit^51^ support the hypothesis that the RSC may be downstream of the hippocampus in the contextual fear memory circuit and plays an important role in information processing within this circuit.

### Localized dendritic plasticity mechanisms link memories

Experience-dependent localized dendritic plasticity has been assessed in ex vivo settings^10,12–15^. However, it was unclear whether new learning induces compartmentalized dendritic plasticity. Moreover, the function of such localized dendritic plasticity in memory processes is unknown. We show that linked contextual memories result in the activation of overlapping dendritic branches within RSC apical dendrites (Fig. 3). The inferred activity rate, a measure in part dependent on the dendritic excitability, was more correlated between segments reactivated following the encoding of linked, but not independent, memories. In addition, we demonstrated that following the formation of linked memories, structural synaptic plasticity (addition of single spines and spine clusters) is also biased to overlapping dendritic segments (Figs. 4 and 5). Although our dendritic calcium imaging results (Fig. 3) did not conclusively rule out a neuron-wide pan-dendritic reactivation, we believe this is unlikely given the low level of dendritic overlap and the wealth of evidence that learning-related plasticity is input specific and only observable on some but not all dendritic/synaptic loci within a neuron^52–55^. Our finding that linked contextual memories are co-allocated to overlapping dendritic branches is consistent with the hypothesis that experience-dependent localized dendritic plasticity is a metaplasticity mechanism that influences future plasticity on these dendritic branches. Indeed, our computational model predicts that a localized increase in dendritic excitability, and the associated facilitation of structural plasticity, are necessary for neuronal overlap during recall (a measure of co-recall in our biophysical model; Fig. 7).

We targeted apical dendrites from layer V RSC neurons as these dendritic compartments undergo clustered plasticity following contextual memory formation^33^. It is currently unclear how the various inputs to RSC dendrites (for example, those from the anterior thalamic nuclei and CA1)^26,27^ interact to facilitate clustered synaptic plasticity and the linking of contextual memories. In addition to the circuit mechanisms, intracellular mechanisms that mediate clustered plasticity are also unknown, but biophysical computational models (Fig. 7) can provide testable hypotheses. Ex vivo investigations of localized dendritic plasticity have revealed several underlying mechanisms including plasticity of dendritic spikes^14,42^, ion channel function^10^, signaling pathways^13^ and protein synthesis-dependent mechanisms^12^. For example, an increase in local dendritic excitability could facilitate dendritic depolarization, which in turn would promote the addition of spine clusters to the same dendritic segment during memory linking.

To demonstrate the critical role of dendritic overlap, we modified the TetTag approach to manipulate specific dendritic segments within tagged RSC cells (Fig. 6). Like the reactivation of neuronal ensembles, the reactivation of RSC dendritic ensembles alone allowed us to extend the temporal window for memory linking. Our results demonstrate that dendritic ensemble overlap plays a key role in memory linking. Given the obvious evolutionary advantage of linking memories that share common elements to the organism’s success, we hypothesize that the mechanisms that allow memory organization are tightly regulated and depend on conserved features across brain regions. Thus, localized dendritic plasticity mechanisms are likely to mediate the organization of memories across various brain regions and various dimensions.

Finally, within the dendritic arbor, inputs from distinct pathways are organized on distinct dendritic domains^56^. Our current findings suggest that only inputs impinging on the same dendritic compartments might be linked, while other inputs remain independent (Fig. 7). Dendritic plasticity mechanisms may facilitate the linking of similar memories (for example, two contextual memories) that recruit inputs synapsing onto overlapping dendritic segments. Memories encoded by inputs synapsing onto non-overlapping dendritic domains do not benefit from dendritic plasticity mechanisms and may remain unlinked. Understanding the roles of branch-specific plasticity mechanisms in differentially modulating inputs within a neuron, and thus the larger neuronal circuit, will be important to understand information processing within a distributed circuit.

## Methods

### Animals

All experimental protocols were approved by the Chancellor’s Animal Research Committee of the University of California, Los Angeles, in accordance with National Institutes of Health guidelines. Adult (3–8 months old) male and female mice were used in the experiments as indicated. cFos-tTa mice were maintained in a C57BL/6N background. Thy1-YFP-H mice (Jackson Laboratories, stock no. 003782) were used for structural imaging experiments. C57BL/6N Tac mice were purchased from Taconic Farms for all other experiments.

### Viral construct

pAAV-Syn-GCaMP6f-WPRE-SV40 was a gift from Douglas Kim & GENIE Project (Addgene viral prep no. 100837-AAV1; RRID: Addgene 100837). The lentivirus hM3Dq-T2A-EGFP vector was derived as previously described in Cai et al.^3^. Finally, AAV1-TRE-hChR2-mCherry, AAV1-TRE-hChR2-mCherry-DTE and AAV1-TRE-mCherry-DTE were derived in our laboratory. Briefly, to construct a vector for TRE-driven hChR2 expression, a CamKIIa promoter from pAAV-CamKIIa-hChR2(H143R)-mCherry (Addgene, 26975) was replaced with TRE promoter from pAAV-RAM-d2tTA∷TRE-NLS-mKate2-WPRE (Addgene, 84474) using MluI/AgeI digestion. The DTE sequence of *Arc* mRNA was PCR amplified from cDNA of 14-week-old Spraque–Dawley rats using primers as previously described^47^ and inserted into the pAAV-TRE-hChR2-mCherry vector using EcoRI/HindIII. The pAAV-TRE-hChR2-mCherry and pAAV-TRE-hChR2-mCherry-DTE were subjected to AgeI/BsrGI digestion for construction of mCherry vectors, respectively. The mCherry was digested by AgeI/BsrGI from pmCherry-N1. Adeno-associated virus (AAV) production was conducted as previously described in detail^57^ with modifications.

### Surgery

Mice were anesthetized with 1.5–2.0% isoflurane for surgical procedures and placed into a stereotaxic frame (David Kopf Instruments) on a heating pad. Artificial tears were applied to the eyes to prevent drying. In addition, carprofen (5 mg per kg body weight) and dexamethasone (0.2 mg per kg body weight) were administered both during surgery and for 2–7 days after surgery. A midline incision was made down the scalp, and a craniotomy was performed with a dental drill. Water with amoxicillin was administered for 2 weeks.

### Miniscope experiments

#### Surgeries

Mice were unilaterally microinjected with 500 nl of AAV1-Syn-GCaMP6f-WPRE-SV40 virus at 20–120 nl min^−1^ into the RSC using the following stereotaxic coordinates: −2.3 mm posterior to bregma, 0.5 mm lateral to the midline and −0.8 mm ventral to the skull surface. Immediately afterward, a 2.0-mm diameter circular craniotomy was centered above the virus injection site. The microendoscope (0.25 pitch, 0.50 NA, 2.0 mm in diameter, 4.79 mm in length, Grintech) was slowly lowered with a stereotaxic arm above the craniotomy 450 μm ventral to skull surface. The microendoscope and a skull screw were fixed with cyanoacrylate and dental cement. Kwik-Sil (World Precision Instruments) was used to cover the microendoscope. Three weeks later, a small aluminum baseplate was cemented onto the animal’s head atop the previously placed dental cement. The microscope was placed on top of the baseplate and locked in a position such that the field of view contained cells and visible landmarks. Finally, a plastic headcap was fit into the baseplate and secured with magnets. Independent experiments confirmed that GCaMP6f expression was limited to RSC neurons (Fig. 1b).

#### Miniscope behavior

Customized UCLA Miniscopes-V3 with a 20-mm achromatic doublet lens and modified housing were used to allow imaging 300 μm below the GRIN lens (allowing imaging of RSC neurons). Before imaging sessions, mice were handled and habituated to the experimental conditions, including carrying the Miniscope. Mice were exposed to each context (with distinct visual, auditory and olfactory cues) for 10 min during which calcium transients were recorded (Fig. 1e). The actual contexts used were counterbalanced and comprised rectangular plastic containers (15 ± 1 by 11 ± 1 inches) covered with various visual cues.

#### Miniscope analysis

Calcium imaging data were registered using NoRMCorre^58^ followed by automated segmentation, demixing and denoising of calcium signals using constrained nonnegative matrix factorization for endoscopic data (CNMFe)^59^. We used a modified version of the Miniscope analysis package developed by G. Etter (Sylvain Williams Lab, McGill University) for data analysis^60^. Recordings from multiple sessions of the same mouse were aligned using an amplitude-based registration algorithm used for within-session registration, except the algorithm was only applied to the mean frame from each session. Once two sessions were registered, ROIs across two sessions were matched to each other using a distance (between ROI centroids) and correlation (between ROIs spatial footprints) measure. The neuronal ensemble overlap was calculated as the percentage of ROIs activated in both contexts divided by the average number of ROIs identified in each imaging session. Neurons were matched across days based on distance (<4 pixels) and correlation (>0.9) thresholds.

In a parallel approach, we aligned and concatenated the imaging data from the three context exposures into a single video file (followed by motion correction and segmentation as described above). The raw data from CNMFe-extracted putative neurons were deconvolved into spike probabilities using the FOOPSI thresholded method (OASIS toolbox). Finally, the spike probabilities from single frames were binarized between 1 (active) and 0 (inactive). For each neuron, the FR (number of active frames per second) for each session was estimated. Population vector correlations were calculated as the Pearson correlation between the average FR (per session) of each neuron across two imaging sessions (Extended Data Fig. 2).

##### NB binary classifier

The activity of each neuron during each 10 min session was resampled into various time bin sizes (0.5–60 s bins, step size of 0.5 s; Extended Data Fig. 2). Resampled data with a specific bin size were used as trials from each session. The classifier was trained on 90% of the data, and we used the information contained in the probability of activity from each neuron to test the remaining 10% of data (tenfold cross-validation strategy) as belonging to the two given sessions. The AUC was calculated for the first context (A for 7 days or B for 5 h; Fig. 1g) using the Wilcoxon–Mann–Whitney statistic. The quality of the classification is defined by the AUC, which ranges from 0 to 1. AUC = ~0.5 means sorting at chance levels by the classifier.

PWC maps for each session were calculated by binning neuronal activity into 100 ms bins to compute the Pearson correlation for each pair of neurons (Supplementary Fig. 1). PWC stability was calculated as the Pearson correlation between PWC maps from different sessions, excluding the main diagonal (correlation between each neuron with itself) and cell pairs below the main diagonal (such that each cell pair was represented only once). Because artificially high correlations can arise due to suboptimal demixing of calcium signals from nearby ROIs, we computed the PWC analysis while ignoring the PWCs from nearby cell pairs (cell pairs where spatial footprints had any overlap or where the centroid–centroid distance was ≤20 pixels (~40 µm)). To control for the different number of neurons detected for different mice, we calculated PWC stability between two sessions by randomly subsampling a group of ten cells, computing the PWC map for each of the sessions using these cells, and computing the Pearson correlation between the two PWC maps. This process was repeated 1,000 times and the final PWC stability was defined as the average of these 1,000 values. The absolute PWC per imaging session and PWC stability across sessions follows the same trend whether the analyses were done with (*t* = 3.4, *P* = 0.009) or without nearby cells or with subsampling of 50 cells instead of 10 cells (*t* = 3.61, *P* = 0.006). For brevity, we only present analyses that excluded the nearby neurons.

### Optogenetic experiments

cFos-tTa transgenic and their wild-type littermates maintained on doxycycline chow (for >1 month) were bilaterally microinjected with 500 nl of AAV1-TRE-hChR2-mCherry, AAV1-TRE-hChR2-mCherry-DTE and AAV1-TRE-mCherry-DTE virus into the RSC. For Extended Data Fig. 6, wild-type mice were injected with a cocktail of CamKII-Cre (Addgene, 105558-AAV; diluted 1:10^3^) with DIO-hChR2 (Addgene, 35509-AAV9; experimental) or DIO-GFP (control). Following viral injections, bilateral optogenetic cannulae (Doric Lenses; DFC_200/240-0.22_0.5mm_GS1.0_FLT) were implanted over the injection site at −0.45 mm ventral to the skull surface.

### Chemogenetic experiments

C57BL/6NTac male mice were bilaterally microinjected with 1,000 nl of lentivirus hM3Dq.T2A.EGFP into the RSC using the following stereotaxic coordinates: −1.95 mm and −2.65 mm posterior to bregma. To ensure that the same RSC neurons are recruited for encoding these different contexts, we transiently increased the intrinsic excitability of a small subset of RSC neurons by administering a clozapine *N*-oxide (0.5 mg per kg body weight) injection 45 min before each context exploration^3^. The control mice only received the clozapine *N*-oxide injection before the second context exploration. Following this, the mice underwent the memory linking paradigm described below.

### Memory linking studies

Linking of context memories was carried out as previously described^3^. Briefly, mice were handled for 3 days (2–5 min per day) and habituated for 3–5 days (2–5 min per day). Mice then explored two distinct contexts (A and B, for 10 min each) separated by 5 h (Fig. 2d and Supplementary Fig. 5) for linking under control conditions or 2 days (Figs. 2e and 6f and Extended Data Figs. 6 and 7) to ensure a robust lack of linking under control conditions^29^. The actual contexts presented were counterbalanced to minimize any effect of context similarity. For Fig. 2d, the context exposure in chamber B also included a 2-s, 0.75-mA footshock that was delivered 58 s before the end of context exposure. This was done to shorten the window of time between the encoding of the first contextual memory (for activity-dependent tagging), subsequent linking and optogenetic manipulation to 24 h after tagging. All optogenetic manipulations were performed 24 h after tagging to ensure sufficient expression of the tagged opsin (Supplementary Fig. 8).

#### Immediate shock

For Figs. 2e and 6f, 2 days following the last context exposure (in B), mice were placed in context B again for an immediate foot shock (10 s baseline, 2 s shock, 0.7–0.75 mA, 28–58-s post-shock period). For Supplementary Fig. 5, to compensate for the lower freezing seen in C57BL/6N Jackson mice (the genetic background of the Thy1-YFP mice), the immediate shock protocol was modified to a 10 s baseline, two shocks for 2 s each, 0.75 mA, 15 s apart.

#### Testing

During the testing phase, mice were tested in the designated contexts (5 min each) on three separate days to minimize any effects of testing animals in one context on subsequent tests in another context. The order of testing was also chosen to control for any gradual increase or decrease in freezing. The actual contexts were counterbalanced.

### Tagging of RSC ensemble

Mice were allowed to recover from surgeries for 3–5 weeks before being handled (3 days) and habituated (3–5 days) for behavioral exposure and optogenetic manipulation. The day after the last day of habituation, mice were taken off doxycycline chow (40 mg per kg body weight) and placed on regular chow and tTA expression was allowed for 3 days before behavioral tagging. The activity-dependent tag was shut off by administration of high doxycycline chow (200 mg per kg body weight) 90 min after behavioral tagging. For experiments in Fig. 2d and Supplementary Fig. 2, a subset of animals was also administered doxycycline intraperitoneally (i.p., 50 μg per gram of body weight; 2 h after tagging) to ensure that the tagging window is closed even in the absence of immediate feeding. The dose and timing were chosen because of its effectiveness in initiating tagging with the Tet-On system^61^ and because doxycycline detection peaks 2 h after injection in the brain tissue^62^. Our behavioral results were the same with and without doxycycline administration i.p.; thus, we combined data from these two sets of experiments in Fig. 2d. In set 1 (without i.p. doxycycline), animals were placed on high doxycycline chow 90 min after context A exposure (control, *n* = 4; TTA-ChR2, *n* = 6; TWRM ANOVA, *F*_Interaction_ (1, 8) = 5.4, *P* < 0.05; Sidak’s test, *P* < 0.005). In set 2 (with i.p. doxycycline), animals were placed on high doxycycline chow and injected with doxycycline i.p. 2 h after context A exposure (control, *n* = 12; TTA-ChR2, *n* = 8; TWRM ANOVA, *F*_Interaction_ (1, 18) = 5.4, *P* < 0.05; Sidak’s test, *P* < 0.005; combined: control, *n* = 16; TTA-ChR2, *n* = 14; TWRM ANOVA, *F*_Interaction_ (1, 28) = 12.8, *P* < 0.005; Sidak’s test, *P* < 0.0001).

### Optogenetic manipulations

All optogenetic manipulations were performed 24 h following the tagging event to ensure sufficient expression of the opsins. For reactivation of tagged ensembles in the home cage (Figs. 2e and 6f), ensembles tagged during the first context exposure were reactivated in the home cage using a 473 nm laser (5 ms pulses, 5 Hz) for 10 min. For testing (Fig. 2c,d), mice were placed in an open field and freezing behavior was recorded using a digital camera. Following a 3 min baseline period, the tagged RSC ensemble was reactivated using a 473 nm laser (5 ms pulses, 5 Hz) for 1 min followed by a 1 min interval with no stimulation. This pattern of stimulation was repeated three times, and the time spent freezing during the three epochs was averaged.

### Immunostaining

Mice were transcardially perfused with 0.1 M phosphate buffer followed by 4% paraformaldehyde. Brains were kept in the fixation solution overnight at 4 °C, transferred to 30% sucrose solution for 48 h, sectioned (40 µm thickness) on a cryostat, and stained while free floating. For staining synaptic proteins, tissue was sectioned at 15 µm in thickness.

The sections were blocked for 1 h at room temperature in 0.3% Triton-X in PBS (PBST) and 10% normal goat serum (Vector Laboratories, S-1000) solution. Primary and secondary antibodies were diluted in the same blocking solution. The primary antibody (guinea pig anti-RFP: SySy 390004 (1:500 dilution); chicken anti-RFP: SySy 409006 (1:500 dilution), anti-PSD-95: SySy 124308 (1:100 dilution), anti-phospho-Cofilin; Millipore C8992 (1:100 dilution) incubation was overnight (~18 h) at 4 °C, and the secondary antibody (Alexa Fluor 488, 568 and 647, Invitrogen; 1:500 dilution) incubation was 2 h at room temperature, both with constant shaking. Immunostaining images were acquired with a Nikon A1 laser scanning confocal microscope and analyzed with an automatic spot-detection algorithm (Imaris 9.2, Bitplane) and manually verified.

### In situ hybridization

Controls were wild-type C57BL/6N mice that were injected with a cocktail of CamKII-Cre (Addgene 105558-AAV1, diluted 1:10^3^) and DIO-GFP viruses to label a sparse and random subset of RSC neurons. The DTE group comprised cFos-tTa mice injected with TRE-Opsin-GFP-DTE (10^11^ genome copies per ml) to sparsely label dendrites in an activity-dependent manner. Mouse brains were dissected and fast frozen in optimal cutting temperature compound using dry ice. Frozen sections were sliced (15 µm). In situ hybridization was performed using the RNAscope Multiplex Fluorescent Reagent Kit v2 (ACD, 323100) with Probe-GFP (ACD, 409011) and Probe-Mm-Arc (316921) per the manufacturer’s instructions.

The images were acquired using NIS-Elements AR (Nikon, v.4.40.00) with a Nikon A1 laser scanning confocal microscope. Analysis of the confocal images was conducted using NIS-Elements AR Analysis software. ROIs were manually delineated to specifically isolate the GFP signal within dendrites (excluding the soma). The GFP and Arc signals within these ROIs were automatically segmented using thresholding techniques. A 1–5-fold dilation of the GFP signal was applied, and the volume of overlap between the dilated GFP signal and the Arc signal was quantified to determine the extent of their colocalization as follows:

$$\begin{array}{l}{\rm{Chance}}\,{\rm{level}}=({\rm{GFP}}\,{\rm{volume}}/{\rm{ROI}}\,{\rm{total}}\,{\rm{volume}})\\\qquad\qquad\qquad\quad\times ({\rm{Arc}}\,{\rm{volume}}/{\rm{ROI}}\,{\rm{total}}\,{\rm{volume}})\end{array}$$

$$\begin{array}{l}{\rm{Arc}}\,{\rm{overlap}}\,{\rm{possibility}}=\left({\rm{GFP}}\,{\rm{and}}\,{\rm{Arc}}\,{\rm{overlap}}\,{\rm{volume}}\right.\\\left.\qquad\qquad\qquad\qquad\qquad\quad/{\rm{ROI}}\,{\rm{total}}\,{\rm{volume}}\right)/{\rm{chance}}\,{\rm{level}}\end{array}$$

### Structural two-photon imaging

Male and female Thy1-YFP-H mice underwent window implantation surgeries as previously described^33^. Briefly, a square 2–3-mm region of the skull was marked (center at RSC: bregma −2.3 mm AP). The skull was thinned and removed. After cleaning the surgical site with saline, a custom-cut sterilized coverslip was placed on the dural surface and fastened with adhesive and dental acrylics to expose a square window of approximately 2 mm. Next, an aluminum bar with a threaded hole was attached to stabilize the mice during imaging. Following recovery from surgery (2–3 weeks), mice were handled and habituated. After handling/habituation (1–2 days later), mice underwent two baseline imaging sessions 2 days apart. Two days later, a subset of mice was exposed to a novel context ‘A’. After 7 days, mice were exposed to two more novel contexts (B and C, 5 h apart). Each context exposure was 10 min and mice were imaged 2 h after each context exposure. Control mice remained in the home cage.

A custom-built two-photon laser scanning microscope with a Spectra-Physics two-photon laser (920 nm) and a 40× 1.0 NA water-immersion objective (Zeiss) was used to acquire images. Mice were lightly anesthetized with isoflurane and head fixed. Segments of apical dendrites from layer V pyramidal cells were acquired within 200 μm from the cortical surface, likely representing dendrites located in layers I and II/III. Imaged segments were generally oriented in the *x*,*y* plane of imaging with minimal *z*-projection. 512 × 512-pixel images were acquired at 0.5 μm intervals to fully capture the segment of dendrite, and image stacks generally consisted of 30–40 slices. The same segments were repeatedly imaged across experimental days by locating their position via a coordinate system established during the first imaging session.

#### Image and data analysis

Dendritic spines were analyzed and counted by established criteria. Specifically, the Spine Analysis software included in ScanImage was used to open all imaging days for a given segment of dendrite. Dependence between new spines added to a dendritic segment following various imaging sessions was calculated using Spearman’s correlation and mutual information. Spearman’s rho (*ρ*) was used as the spine addition/loss data did not follow a normal distribution. For mutual information analysis, statistical significance was calculated by comparing the observed value to the *z*-score of the chance distribution. A distribution of chance values was calculated by randomly permuting the number of spines added during the second imaging session (10,000×).

Clustering ratios were calculated as the number of clustered spines divided by the total number of new spines gained between two time points. Clustered spines were defined as a new spine that was less than 5 µm from another new spine. For the resampling analysis of clustering, the number of new spines added per segment of dendrite was used to pick an equivalent number of random positions along the same segment (regardless of whether a spine was recorded on that spot on a previous imaging session) and assess whether these positions were within 5 μm of each other. When this was completed for all dendrites for a given animal, the percentage of clustered spines was calculated as the number of randomly selected new spine positions within 5 μm of each other divided by the total number of stably added new spines for that animal. In turn, each animal’s resampled clustering percentage was calculated, and then these values were averaged together. This completed one resampling event, and this process was then repeated 1,000×, which then yielded the full distribution of random sampling (Extended Data Fig. 9). New spine formation following learning was correlated with spine density in control conditions (*P* = 0.007) but not following a context exposure. Spine formation or clustering was not correlated with pre-learning turnover (all *P* values are not significant).

#### Cross-clustering across exposures

The clustered spines added following a context exposure were randomly distributed on the dendritic segments from that mouse (10,000×). The percentage of clustered spines added to a dendritic segment following the first context exposure, which were added to a segment that also gained clustered spines following the subsequent context exposure, was measured and compared to the shuffled distribution obtained from the above analysis. The distance between two newly formed spines following each imaging session was calculated for spine pairs that were the nearest neighbors. If no new spine was added or no newly formed spines persisted during the final imaging session (reference session), these dendrites were not considered during the analysis. Our results remained the same when dendrites with non-persistent or no newly added spines are included in the analysis (5 h: 32.1%, average distance between nearest neighbors = 18.1 μm ± 2.2 μm; 7 days: 11.1%, average distance between nearest neighbors = 30.9 μm ± 2.3 μm; *P* < 0.0001). In this case, the length of the dendritic segment was considered the average distance between nearest neighbors.

#### Resampling analysis

For analysis performed in Fig. 5e–g, dendritic branches (*n* = 40) from each condition were subsampled (10,000×) to obtain cumulative frequency distributions for Spearman correlations, mutual information and spine clustering probability for each condition. Insets demonstrate the difference between observed measurements for each variable from context exposure and HC groups imaged at the 5 h interval. *P* values were calculated as: (number of measurements where the difference between experimental versus control group < 0/10,000).

### Functional two-photon imaging

Mice underwent bilateral injection of GCaMP6f (final concentration ~10^11^ viral genomes per ml) in the RSC to achieve semi-sparse infection of layer V RSC neurons^63^. All dendritic imaging experiments were completed within 25 days of virus injection to prevent viral overexpression. A square 3 mm × 3 mm craniotomy spanning the midline, and hence revealing both RSCs, was then made over the injection. Two to three weeks following the surgery, mice underwent handling (3 days) and habituation (3 days) to acclimate to the treadmill and head fixation. Neuronal and dendritic calcium activity was imaged in head-fixed mice that were free to run on a head-fixed setup.

We recorded dendritic signals evoked spontaneously and during context exposure using a resonant-scanning two-photon microscope (Neurolabware) controlled by Scanbox acquisition software. Distinct contexts were created by immobilizing the mice on a running wheel, a treadmill or a horizontal disc (Supplementary Fig. 3), in addition to distinct auditory, olfactory and visual cues associated with each context. Visual stimuli were presented on a large LCD monitor directly in front of the animal. Visual stimuli consisted of non-repeating natural movies with intermittent gray screens (9 s on, 14 s off). Spontaneous response data were collected with a blank gray screen in the absence of auditory and olfactory cues. A Coherent Discovery laser (Coherent) was used for GCaMP excitation, fixed at a wavelength of 920 nm. The objective used was a ×16 water-immersion lens (Nikon, 0.8 NA, 3 mm working distance). Image sequences were captured at 15.5 Hz at a depth of 30–50 µm below the brain surface for apical tuft dendrites and 320–450 µm for layer V RSC neurons in separate animals.

Collected data were processed using the Suite2P analysis pipeline^64^. Recorded frames were aligned using a nonrigid motion-correction algorithm. Following alignment, any frames with significant motion in the *z* axis were dropped from the original video and the data were reanalyzed. ROIs (representing dendritic segments) were segmented in a semiautomated manner using a Suite2p-based classifier. Dendritic segments were matched across imaging sessions using an open-source algorithm (https://github.com/ransona/ROIMatchPub/; matching criterion: correlation of 0.4). The percentage of reactivated dendrites was defined as the number of matched segments normalized to the average number of dendritic segments detected in each imaging session.

### Hierarchical clustering of dendritic ROIs

To merge any dendritic ROI with highly correlated calcium transients into a single dendritic segment, we adapted a hierarchical clustering method^65^ previously used to assign axonal boutons to the same source with some variations. Briefly, we generated a sparse activity matrix by thresholding calcium transients from each ROI such that only frames with activity three standard deviations above the mean activity were retained. The time course of calcium transients for each ROI was then cross-correlated with all other ROIs during the same session to generate a matrix of Pearson correlation coefficients between all ROI pairs. This matrix was thresholded in two ways to obtain a sparse matrix. Only those correlation coefficients > 0.7 or > 2.5 standard deviations above the mean value of all the coefficients between this ROI and all others were used. If neither of these conditions was met for a given ROI pair, the associated correlation coefficient was set to 0. The cosine similarity between every ROI pair was then computed from the thresholded matrix of Pearson correlation coefficients.

Next, we classified ROIs with similar activities into clusters using agglomerative hierarchical clustering based on the pairwise distance, computed as ‘1 − cosine similarity’ and the weighted-pair group method with arithmetic means algorithm. To choose a distance cutoff at which ROIs were considered in the same cluster (or the same dendritic segment), we generated a correlation matrix using a shuffled distribution for each animal. The time course of calcium activity from each ROI from each mouse was circularly shuffled by a random amount. This procedure essentially ensures uncorrelated activity in all ROIs, and the cutoff value that yielded at least one inaccurate cluster in less than 5% of the trails (500 trails) was used as the cutoff for that animal (mean cutoff value = 0.13 ± 0.01). To ensure that clustering criteria were not lenient, we also used singular cutoff values—0.15 or 0.3—to cluster less-correlated ROIs. When used for all animals, these criteria showed similar results (*P* < 0.001). The clustering method yielded ROI clusters with highly correlated within-cluster activity across sessions (reference and comparison sessions for reactivated ROIs; Supplementary Fig. 4). The clustered ROIs within reactivated segments maintained high within-cluster correlated activity across sessions, demonstrating the robustness of our clustering algorithm and the longitudinal coupling of these ROIs (Supplementary Fig. 4c).

For analysis of correlated dendritic activity, dendritic activity/events were estimated from Suite2p-extracted signal using the Vanilla algorithm^63,66^. Event probabilities were binarized and the number of active frames was used to calculate an event rate. To account for variations in the number of reactivated ROIs in imaging sessions 5 h and 7 days apart, we randomly subsampled 30 reactivated ROI pairs for each comparison (500×) to generate a probability distribution.

### Whole-cell patch recordings

The brain was rapidly dissected out and transferred to oxygenated (95% O_2_/5% CO_2_), ice-cold cutting solution containing: 92 mM choline, 2.5 mM KCl, 1.2 mM NaH_2_PO_4_, 30 mM NaHCO_3_, 20 mM HEPES, 25 mM glucose, 2 mM thiourea, 5 mM Na-ascorbate, 3 mM Na-pyruvate, 5 mM *N*-acetyl-l-cysteine, 0.5 mM CaCl_2_ and 10 mM MgSO_4_. RSC coronal slices (300 µm thick) were cut using a Leica VT1200, transferred to a submerged holding chamber containing oxygenated cutting solution and allowed to recover for 15 min at 34 °C. Following recovery, the slices were transferred to an oxygenated solution containing: 92 mM choline, 2.5 mM KCl, 1.2 mM NaH_2_PO_4_, 30 mM NaHCO_3_, 20 mM HEPES, 25 mM glucose, 2 mM thiourea, 5 mM Na-ascorbate, 3 mM Na-pyruvate, 5 mM *N*-acetyl-l-cysteine, 2 mM CaCl_2_ and 2 mM MgCl_2_ and allowed to recover for 1 h. Following incubation, slices were transferred to a recording chamber and constantly perfused with oxygenated artificial cerebrospinal fluid containing 115 mM NaCl, 10 mM glucose, 25.5 mM NaHCO_3_, 1.05 mM NaH_2_PO_4_, 3.3 mM KCl, 2 mM CaCl_2_ and 1 mM MgCl_2_ and maintained at 28 °C. For two mice (TTA-ChR2 and TTA-ChR2-DTE, *n* = 1 each), brains were sliced in recording solution.

Whole-cell current-clamp recordings were performed as previously described^67^. All recordings were obtained using a MultiClamp 700B amplifier controlled by the pClamp 10 software and digitized using the Digidata 1440A system. Signals were filtered at 10 kHz and digitized at 20 kHz. Neurons were included if the initial resting membrane potential (Vm) ≤ −55 mV, access resistance (Ra) was <25 MΩ and were rejected if the Ra changed by >20% of its initial value. For all recordings, neurons were held at −60 mV. To investigate the response of the neurons following optogenetic stimulation, a 473 nm LED (5 ms pulses, 5 Hz) was delivered through Cool LED pE-300 and neuronal response was calculated. Only neurons with a visible response to optogenetic stimulation were included in the analysis (*n* = 12 from >50 RSC neurons). All mCherry-positive RSC neurons from TTA-ChR2 mice resulted in action potentials following stimulation.

### Computational modeling

We adapted a previously published model network of memory allocation and excitability^32^. Neurons consist of a somatic spiking unit connected to multiple independent dendritic subunits. Inhibitory neurons are separated into soma-targeting and dendrite-targeting neurons. Dendritic voltage was as given by equation (1):

1 $${C}\frac{{\rm{d}}{{V}}_{{\rm{d}}}}{{\rm{d}}{t}}\,=\,-{{g}}_{{\rm{L}}}\left({{V}}_{{\rm{d}}}-{{E}}_{{\rm{L}}}\right)+\,{{a}}_{{\rm{exc}}}{{g}}_{{\rm{E}}}{{u}}_{{\rm{E}}}\left({t}\right)\left({{E}}_{{\rm{E}}}-{{V}}_{{\rm{d}}}\right)-{{g}}_{{\rm{I}}}{{u}}_{{\rm{I}}}({t})\left({{E}}_{{\rm{I}}}-{{V}}_{{\rm{d}}}\right)$$

where *V*_d_ is the dendritic voltage, *C* is membrane capacitance, *E*_E_ and *E*_I_ are the excitatory and inhibitory reversal potential, *E*_L_ is the resting potential (0 mV), *a*_exc_ is dendritic excitability parameter, *g*_L_ is leak conductance, and *g*_E_ and *g*_I_ are the maximal excitatory and inhibitory synaptic conductances. *u*_I_(*t*) and *u*_E_(*t*) are instantaneous activations of excitatory and inhibitory synapses on the dendrite according to equation (2):

2 $${u}_{{\rm{E}}/{\rm{I}}}\left({t}\right)={\sum }_{{j}}{{w}}_{{j}}{\rm{\delta }}\left({t}-{{t}}_{{j}}\right)$$

where *w*_*j*_ is the weight of synapse *j* and *t*_*j*_ are the timings of incoming spikes.

Somatic voltage follows the integrate-and-fire model with adaptation dynamics according to equations (3)–(6):

3 $${C}\frac{{\rm{d}}{V}}{{\rm{d}}{t}}=\,-{{g}}_{{\rm{L}}}\left({V}-{{E}}_{{\rm{L}}}\right)+{{I}}_{{\rm{noise}}}\left({t}\right)+{{I}}_{{\rm{ax}}}\left({t}\right)-{{I}}_{{\rm{inh}}}\left({t}\right)-\,{{I}}_{{\rm{adapt}}}\left({t}\right)$$

4 $${{\rm{\tau }}}_{{\rm{adapt}}}\frac{{\rm{d}}{{I}}_{{\rm{adapt}}}}{{\rm{d}}{t}}={{\rm{\alpha }}}_{{\rm{adapt}}}\left({V}-{{E}}_{{\rm{L}}}\right)+\,{\beta}_{{\rm{adapt}}}{\rm{\delta }}({t}-{{t}}_{{\rm{spike}}})-\,{{I}}_{{\rm{adapt}}}$$

5 $${{I}}_{{\rm{ax}}}={\sum}_{{\rm{i}}}{{g}}_{{\rm{ax}}}({V}_{{\rm{d}},{\rm{i}}}-{V})_{+}$$

6 $${{\rm{\tau }}}_{{\rm{inh}}}\frac{{{\rm{d}}{I}}_{{\rm{inh}}}}{{\rm{d}}{t}}=\,{\sum }_{{\rm{i}}}{{g}}_{{\rm{inh}}}{\rm{\delta }}({t}-{{t}}_{{\rm{i}}})-{{I}}_{{\rm{inh}}}$$

*V* is the somatic voltage, *I*_noise_ is uniform noise current (maximum 500 pA); *I*_ax_ is the excitatory axial current; *I*_inh_ is the filtered inhibitory current of soma-targeting interneurons; *I*_adapt_ is the adaptation current; *τ*_adapt_ is the adaptation time constant; *α*_adapt_ is the adaptation coupling parameter; *β*_adapt_ is the adaptation step per spike; *g*_ax_ is the axial resistance; *τ*_inh_ is the inhibitory time constant; and *g*_inh_ is the inhibitory parameter. Incoming spikes increase synaptic and dendritic branch calcium by ΔCa(*V*_d_) accounting for magnesium blocking of NMDA receptors as given by equation (7):

7 $$\Delta {\rm{Ca}}\left({{V}}_{{\rm{d}}}\right)=\frac{1}{1+\frac{{{\rm{e}}}^{-0.07\left({{V}}_{{\rm{d}}}-70\right)}}{9}}$$

Memory synapses target random dendrites with weights between 0.16 and 0.36 (Supplementary Table 2). During dendritic separation (Fig. 7g), memories were not allowed to overlap in the same dendrite.

To replicate the finding that new synapses correlate with the potentiated ones, synapses that receive calcium influx > 0 became candidates for potentiation or depression. If the neuron has ff > 10 Hz during a stimulus, the synapse is tagged for potentiation with probability *p*_LTP_ = 0.29 *+* *X*_dend_ × *N*_s_/2; otherwise the synapse is tagged for depression (*N*_s_ denotes preexisting potentiated synapses; *X*_dend_ denotes dendritic excitability; LTP denotes long-term potentiation).

If neuronal calcium level exceeds *Θ*_PRP_ at time *T* (min), a PRP transient occurs according to equation (8):

8 $${\rm{PRP}}\left({T}\right)=\left\{\begin{array}{c}\frac{{T}-20}{30}{{\rm{e}}}^{-\frac{{T}+10}{30}},{ T} > 20\\ 0,{ T}\le 20\end{array}\right.$$

Synapse weights are updated by Δ*w* = 0.15 × PRP(*t*) × (synaptic tag), where *t* is in seconds, when tag > 0.1 and plasticity-related proteins (PRP) > 0.1. Weights were clipped to [0, 1]. Due to the 5 h delay between memories, there was no competition for PRPs. A branch was considered to contain overlapping memory clusters if it contained at least three potentiated synapses from each memory.

#### Excitability within the linking model

When total dendritic and somatic calcium are above thresholds *Θ*_dend_ and *Θ*_soma_, respectively, at time *T* (hours), the excitability level *Χ* of the dendrite or soma is as given by equation (9):

9 $${{X}}_{{\rm{dend}},{\rm{soma}}}({T})=\frac{1}{1+{{\rm{e}}}^{-3\left({T}-1\right)}}-\,\frac{1}{1+{{\rm{e}}}^{-\left({T}-26\right)}}$$

In addition, *a*_exc_ increases by 10% when *X*_dend_ > 0, and *β*_adapt_ increases by 28% when *X*_soma_ > 0.1. For the linking model without dendritic mechanisms, *X*_dend_ = 0 and *p*_LTP_ = 0.32.

Synaptic weights *w*_*j*_ follow homeostatic synaptic scaling with time constant *τ*_H_, as given by equation (10):

10 $$\frac{{\rm{d}}{{w}}_{{j}}}{{\rm{d}}{t}}\,=\,\frac{1}{{{\rm{\tau }}}_{{\rm{H}}}}\left(1{{-}}\frac{{\sum }_{{j}}{{w}}_{{j}}}{{{w}}_{{\rm{init}}}{{N}}_{{\rm{syn}}}}\right)$$

*w*_init_ = 0.3, and *N*_syn_ is the total synapses to the neuron.

#### Stimulation protocol

For memory, the synaptic inputs are firing for 4 s at 35 Hz. Then, a delay of 5 h or 2 days or 7 days is simulated, the second memory is encoded and memories are recalled after a delay of 2 days.

The parameters of the model are listed in Supplementary Table 2. The source code is available at https://dendrites.gr/wp-content/uploads/2022/08/rsc_model2.zip.

Chance levels for neuronal overlap were calculated as previously described^3^: Chance overlap = [(neuronal ensemble encoding A × neuronal ensemble encoding B)/100]. Percentage above chance overlap = (observed overlap − chance overlap)/chance overlap.

### Statistics and reproducibility

The investigator who collected and analyzed the data including behavior, imaging and staining was blinded to the mouse genotypes and treatment conditions. No statistical methods were used to predetermine sample sizes but our sample sizes are similar to those reported in previous publications^3,29^. When appropriate, animals were assigned to groups using a random number generator and experimental conditions were counterbalanced to ensure experimental groups were distributed evenly throughout the experimental timeline. All data shown in column and line graphs represent the mean ± s.e.m., unless otherwise mentioned. All statistical analyses were performed using GraphPad Prism 9 or MATLAB. For behavior and imaging experiments, *n* designates the number of mice unless otherwise mentioned. Statistical significance for behavioral manipulations was assessed using parametric tests (Student’s *t*-test, or one-way or two-way ANOVA) followed by the indicated post hoc tests (GraphPad Prism 9 recommended post hoc tests) as data followed a Gaussian distribution. Nonparametric tests (Kolmogorov–Smirnov and Mann–Whitney) were used to analyze in vivo imaging data where assumption of normality was not met. The level of significance was set at *P* < 0.05 unless Bonferroni’s correction for multiple comparisons was used. Significance levels are indicated as *P < 0.05, **P < 0.01, ***P < 0.001 and ****P < 0.0001. For experiments depicted in Figs. 1e, 2d,e, 3a, 4b and 6c,d,f, a minimum of two experimental cohorts were used. Representative histological images were repeated independently in different mice with similar results for Figs. 2b and 6b (*n* = 4 per group) and Extended Data Fig. 6 (*n* = 7 per group).

Mice were excluded from behavioral experiments (memory linking experiments) if freezing during pre-shock exposures was >20%. For electrophysiology experiments, neurons were excluded per the criteria described in ‘Whole-cell patch recordings’.

### Reporting summary

Further information on research design is available in the Nature Portfolio Reporting Summary linked to this article.

## Online content

Any methods, additional references, Nature Portfolio reporting summaries, source data, extended data, supplementary information, acknowledgements, peer review information; details of author contributions and competing interests; and statements of data and code availability are available at 10.1038/s41593-025-01876-8.

## Supplementary information

Supplementary Information Supplementary Figs. 1–8 and Tables 1 and 2.

Reporting Summary

Supplementary Video 1 Two-photon calcium imaging with branch-specific activity.

## Source data

Source Data Fig. 1 Statistical source data.

Source Data Fig. 2 Statistical source data.

Source Data Fig. 3 Statistical source data.

Source Data Fig. 4 Statistical source data.

Source Data Fig. 5 Statistical source data.

Source Data Fig. 6 Statistical source data.

Source Data Fig. 7 Statistical source data.

Source Data Extended Data Fig. 1 Statistical source data.

Source Data Extended Data Fig. 2 Statistical source data.

Source Data Extended Data Fig. 3 Statistical source data.

Source Data Extended Data Fig. 4 Statistical source data.

Source Data Extended Data Fig. 5 Statistical source data.

Source Data Extended Data Fig. 6 Statistical source data.

Source Data Extended Data Fig. 7 Statistical source data.

Source Data Extended Data Fig. 8 Statistical source data.

Source Data Extended Data Fig. 9 Statistical source data.

Source Data Extended Data Fig. 10 Statistical source data.

## Data Availability

The original videos and datasets generated during and/or analyzed during the current study are available from the corresponding authors. Source data are provided with this paper.

## Code availability

The MATLAB code used for the data analysis is available as a GitHub repository (https://github.com/Almeida-FilhoDG/ConcatMiniscope/).
