# Supplementary material for: Compartmentalized dendritic plasticity in the mouse retrosplenial cortex links contextual memories formed close in time
Source: Nat Neurosci. 2025 Feb 17;28(3):602–15. doi: 10.1038/s41593-025-01876-8 (PMC11893454; doi:10.1038/s41593-025-01876-8)
Supplement: Supplementary file 1 — Supplementary Figs. 1–8 and Tables 1 and 2. [file 41593_2025_1876_MOESM1_ESM.pdf]

nature neuroscience

Supplementary information https://doi.org/10.1038/s41593-025-01876-8

Compartmentalized dendritic plasticity in the mouse retrosplenial cortex links contextual memories formed close in time

In the format provided by the authors and unedited

Supplementary Table 1

Centroid Distance

SFP correlation	3	4	5	6	7	8	9
**0.6**	**7d**: 26.0 ± 4.8<br>**5h**: 50.6 ± 5.3<br>p = 0.0005	**7d**: 37.0 ± 5.4<br>**5h**: 59.7 ± 5.3<br>p = 0.002	**7d**: 46.8 ± 5.2<br>**5h**: 66.1 ± 4.1<br>p = 0.002	**7d**: 51.3 ± 5.0<br>**5h**: 70.2 ± 3.3<br>p = 0.0007	**7d:** 54.3 ± 4.6<br>**5h:** 72.6 ± 2.8<br>p = 0.0003	**7d:** 57.5 ± 4.3<br>**5h:** 74.5 ± 2.6<br>p = 0.0003	**7d:** 59.1 ± 4.2<br>**5h:** 75.3 ± 2.6<br>p = 0.0005
**0.65**	**7d**: 25.4 ± 4.6<br>**5h**: 50.1 ± 5.2<br>p = 0.0005	**7d**: 35.8 ± 5.3<br>**5h**: 58.8 ± 5.2<br>p = 0.001	**7d**: 44.7 ± 5.2<br>**5h**: 64.7 ± 4.1<br>p = 0.001	**7d**: 48.8 ± 5.0<br>**5h**: 68.6 ± 3.2<br>p = 0.0004	**7d:** 51.1 ± 4.7<br>**5h:** 70.5 ± 2.9<br>p = 0.0003	**7d:** 53.1 ± 4.5<br>**5h:** 72.0 ± 2.7<br>p = 0.0002	**7d:** 54.3 ± 4.5<br>**5h:** 72.6 ± 2.7<br>p = 0.0003
**0.7**	**7d**: 24.5 ± 4.5<br>**5h**: 49.3 ± 5.3<br>p = 0.0004	**7d**: 34.0 ± 5.1<br>**5h**: 57.9 ± 5.1<br>p = 0.0007	**7d**: 41.3 ± 5.1<br>**5h**: 63.3 ± 4.3<br>p = 0.0009	**7d**: 44.7 ± 4.9<br>**5h**: 66.4 ± 3.8<br>p = 0.0003	**7d:** 46.3 ± 4.7<br>**5h:** 67.9 ± 3.3<br>p = 0.0002	**7d:** 47.8 ± 4.7<br>**5h:** 69.1 ± 3.0<br>p = 0.0001	**7d:** 48.8 ± 4.6<br>**5h:** 69.5 ± 3.0<br>p = 0.0002
**0.75**	**7d**: 23.5± 4.3<br>**5h**: 47.4 ± 5.2<br>p = 0.0004	**7d**: 31.3 ± 4.9<br>**5h**: 55.5 ± 5.1<br>p = 0.0005	**7d**: 37.1 ± 5.0<br>**5h**: 59.8 ± 4.7<br>p = 0.0006	**7d**: 39.6 ± 4.8<br>**5h**: 62.3 ± 4.5<br>p = 0.0003	**7d:** 41.0 ± 4.7<br>**5h:** 63.6 ± 4.1<br>p = 0.0002	**7d:** 41.9 ± 4.7<br>**5h:** 64.6 ± 3.8<br>p = 0.0002	**7d:** 42.3 ± 4.7<br>**5h:** 64.9 ± 3.9<br>p = 0.0002
**0.8**	**7d**: 21.7 ± 4.1<br>**5h**: 45.0 ± 5.2<br>p = 0.0006	**7d**: 27.8 ± 4.7<br>**5h**: 52.1 ± 5.2<br>p = 0.0007	**7d**: 31.8 ± 4.8<br>**5h**: 55.8 ± 5.0<br>p = 0.0005	**7d**: 33.4 ± 4.7<br>**5h**: 57.7 ± 4.8<br>p = 0.0003	**7d:** 34.2 ± 4.7<br>**5h:** 58.3 ± 4.7<br>p = 0.0003	**7d:** 34.5 ± 4.7<br>**5h:** 58.8 ± 4.6<br>p = 0.0002	**7d:** 34.8 ± 4.7<br>**5h:** 58.9 ± 4.6<br>p = 0.0002
**0.85**	**7d**: 17.8 ± 4.0<br>**5h**: 39.9 ± 5.0<br>p = 0.0008	**7d**: 22.2 ± 4.6<br>**5h**: 44.8 ± 5.2<br>p = 0.001	**7d**: 24.2 ± 4.7<br>**5h**: 47.3 ± 5.0<br>p = 0.0009	**7d**: 24.9 ± 4.6<br>**5h**: 48.5 ± 4.9<br>p = 0.0006	**7d:** 25.3 ± 4.6<br>**5h:** 48.8 ± 4.8<br>p = 0.0006	**7d:** 25.5 ± 4.6<br>**5h:** 49.2 ± 4.8<br>p = 0.0005	**7d:** 25.7 ± 4.6<br>**5h:** 49.3 ± 4.7<br>p = 0.0005
**0.9**	**7d**: 11.9 ± 3.1<br>**5h**: 31.8 ± 4.5<br>p = 0.001	**7d**: 13.9 ± 3.3<br>**5h**: 34.5 ± 4.6<br>p = 0.0008	**7d**: 14.6 ± 3.4<br>**5h**: 35.7 ± 4.5<br>p = 0.0007	**7d**: 14.8 ± 3.4<br>**5h**: 36.2 ± 4.4<br>p = 0.0006	**7d:** 15.0 ± 3.4<br>**5d:** 36.4 ± 4.4<br>p = 0.0006	**7d:** 15.1 ± 3.4<br>**5h:** 36.6 ± 4.3<br>p = 0.0005	**7d:** 15.1 ± 3.4<br>**5h:** 36.7 ± 4.3<br>p = 0.0005
**0.95**	**7d**: 3.9 ± 1.1<br>**5h**: 15.3 ± 3.3<br>p = 0.003	**7d**: 4.2 ± 1.1<br>**5h**: 15.8 ± 3.5<br>p = 0.004	**7d**: 4.2 ± 1.1<br>**5h**: 16.1 ± 3.4<br>p = 0.003	**7d**: 4.3 ± 1.1<br>**5h**: 16.2 ± 3.4<br>p = 0.003	**7d:** 4.3 ± 1.1<br>**5d:** 16.2 ± 3.4<br>p = 0.003	**7d:** 4.3 ± 1.1<br>**5h:** 16.2 ± 3.4<br>p = 0.003	**7d:** 4.3 ± 1.1<br>**5h:** 16.4 ± 3.4<br>p = 0.003

Supplementary Table 2

Parameters of the computational model		
N_{pyr}	Number of excitatory neurons	400
N_{inh}	Number of inhibitory neurons	50 dendrite-targeting (DT)<br>50 soma-targeting (ST)
N_{dend}	Number of dendritic subunits per neuron	10 for excitatory<br>1 for interneurons
$N_{pyr \rightarrow ST}$	Synapses from excitatory neurons to soma-targeting(ST) interneurons	Count: 1000<br>Weight: 0.6
$N_{pyr \rightarrow DT}$	Synapses from excitatory neurons to dendrite-targeting (DT)	Count: 1000<br>Weight: 0.3
$N_{ST \rightarrow pyr}$	Synapses from ST interneurons to excitatory neurons	Count: 10000<br>Weight: 0.5
$N_{DT \rightarrow pyr}$	Synapses from DT interneurons to excitatory neurons	Count: 2000<br>Weight: 0.3
$N_{input \rightarrow pyr}$	Synapses from input afferents to pyramidal dendrites per encoded memory	Count: 23000<br>Initial Weight: 0.16 – 0.36
E_L	Leakage reversal potential	0 mV
g_E / g_I	Dendritic excitatory / inhibitory synaptic conductance	22nS / 20nS
g_{Ld} / g_L	Dendritic/somatic leak conductance	10nS / 8nS
g_{Inh}	Somatic inhibitory current scaling constant	600nS
τ_{Inh}	Somatic inhibitory current time constant	30msec
E_E / E_I	Excitatory /inhibitory synapse reversal potential	+70mV / -10mV
C	Membrane capacitance	200pF
τ_{dend}	Dendritic membrane time constant	Inhibitory: 20msec<br>Excitatory: 25msec
V_d	Dendritic Depolarization	$-10mV < V_d < 70mV$
g_{ax}	Axial conductance	36nS
θ_{soma}	Voltage threshold for somatic spikes	18mV
τ_{adapt}	Adaptation time constant of excitatory neurons	200msec
β_{adapt}	Adaptation reset constant	Baseline excitability: 9<br>High excitability: 6.5
a_{adapt}	Adaptation coupling parameter	0.02
Θ_{PRP}	Calcium threshold for somatic Plasticity-Related Protein (PRP) synthesis	40.0
Θ_{dend}	Calcium threshold for dendritic excitability	2.0
Θ_{soma}	Calcium threshold for somatic excitability	40.0
τ_H	Time constant of homeostatic synaptic scaling	1440 hours

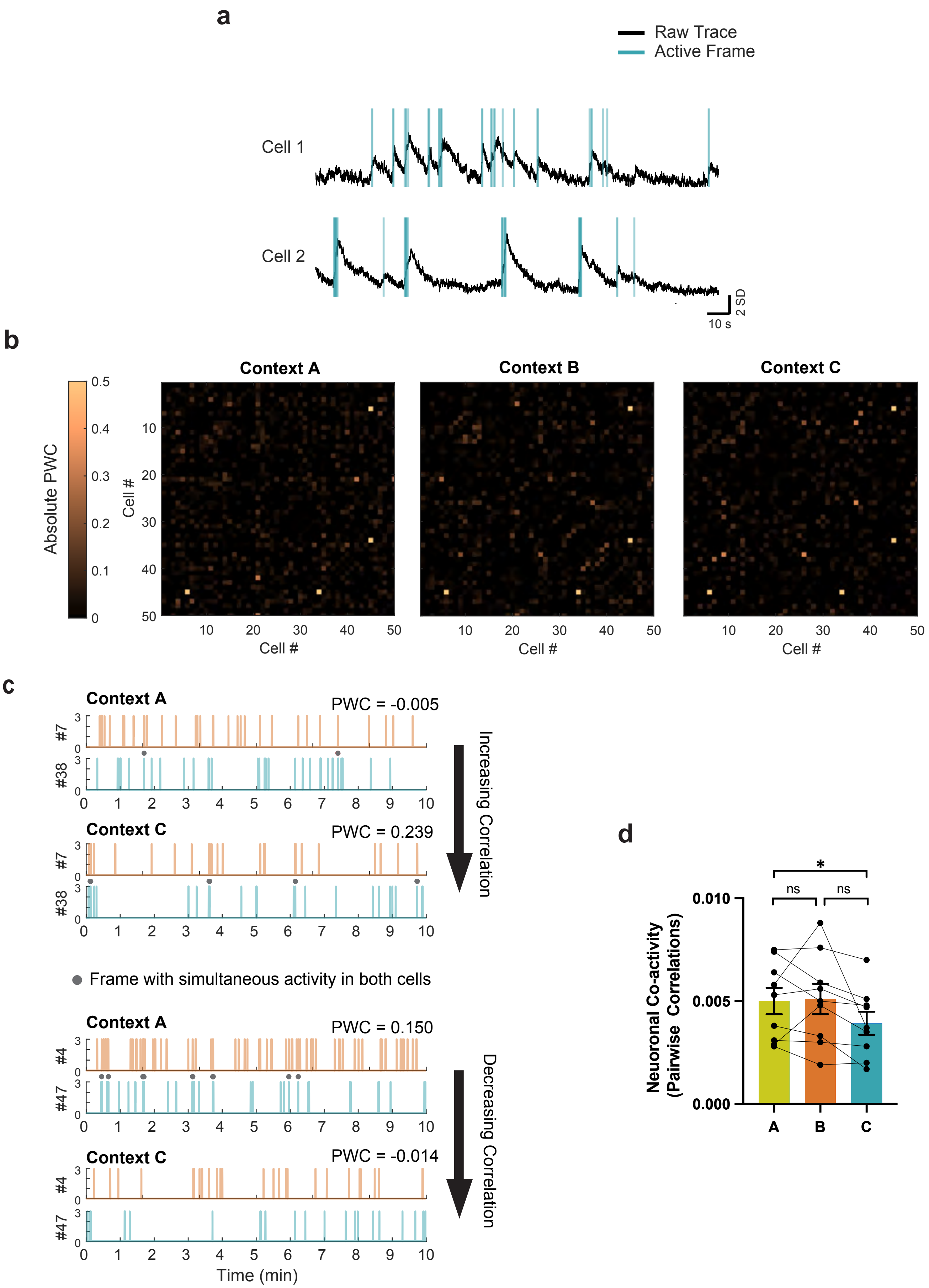

a
Raw Trace
Active Frame
Cell 1
Cell 2
2 SD
10 s
b
Context A
Context B
Context C
Absolute PWC
Cell #
Cell #
c
Context A
PWC = -0.005
#7
#38
Context C
PWC = 0.239
Increasing Correlation
Frame with simultaneous activity in both cells
Context A
PWC = 0.150
#4
#47
Context C
PWC = -0.014
Decreasing Correlation
Time (min)
d
Neuoronal Co-activity (Pairwise Correlations)
ns
ns
*
A
B
C

Supplementary Figure 1. Coactivity among RSC neurons during multiple contextual exposures.

(a) Example of two co-active neurons: For each imaging session, deconvolved neuronal activity was binned into 100ms periods, and the Pairwise correlation (PWC: Pearson correlation of the number of active frames) for each neuron pair was calculated. Black: raw calcium trace and Blue: frames identified as active following deconvolution of the calcium trace.

(b) Pairwise Correlation (PWC) map for each context for 50 cell pairs from one mouse. Absolute Pearson's correlation coefficients were plotted.

(c) Top Panel: Example of a neuronal pair with increasing PWC between imaging sessions: Cell pair (7 and 38: arbitrary cell IDs) display more coactivity (number of simultaneous active frames) during Context C exploration than Context A exploration. Bottom Panel: Example of a neuronal pair with decreasing PWC between imaging sessions: Cell pair (4 and 47: arbitrary cell IDs) display less coactivity during Context C exploration than Context A exploration.

(d) Average PWCs across the three context exposures display a small decrease (One-way repeated measure ANOVA; $F(1.6, 12.9) = 4.05$, $p = 0.05$; Tukey's post-hoc test, n = 9 mice per group).

Data represent mean ± s.e.m. and each data point, * $p < 0.05$.

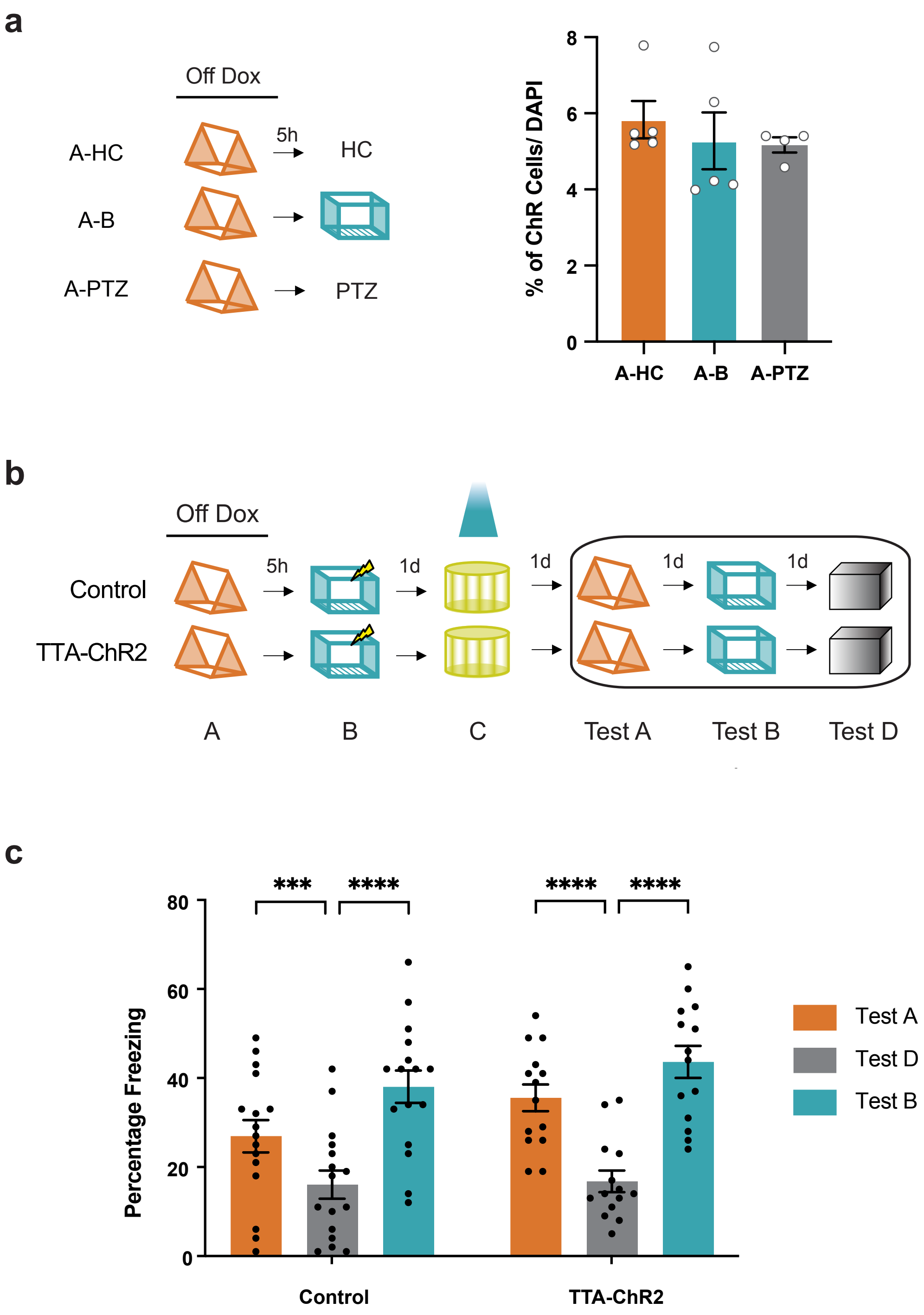
a
Off Dox
A-HC
5h
HC
A-B
A-PTZ
PTZ
% of ChR Cells/ DAPI
8
6
4
2
0
A-HC
A-B
A-PTZ
b
Off Dox
Control
5h
1d
1d
1d
1d
TTA-ChR2
A
B
C
Test A
Test B
Test D
c
***
****
****
****
Percentage Freezing
80
60
40
20
0
Control
TTA-ChR2
Test A
Test D
Test B

Supplementary Figure 2. Fear expression due to reactivation of the linked memory ensemble is not driven by changes in the strength of memory linking or tagging of cells outside of the first behavioral episode.

(a) Five hours after context exposure, the tagging window is closed by doxycycline. RSC ensemble activated during the exploration of the first context (context A) was tagged. The tagging window was closed by placing the mice on a high concentration of doxycycline chow (200mg/kg) as well as a doxycycline injection (i.p., 50 μg/ gram of body weight) two hours post exposure to context A. Five hours following the context exposure mice were either left in home cage (A-HC), explored another context (A-B) or received an injection of pentylenetetrazole (PTZ, 30mg/kg, A-PTZ). Animals were perfused and brains collected 24 hours post-tagging (same timepoint as optogenetic activation for experiment in Figure 2d). No differences in Channelrhodopsin/mCherry expression were observed between groups (One-way ANOVA, F (2, 11) = 0.4, $p = 0.7$; Dunnett's multiple comparisons test, ns; n = 5, 5, 4 for A-HC, A-B, A-PTZ groups).

(b) Following optogenetic activation of the first memory ensemble (Figure 2d), mice were tested in the linked context (Context A), shock context (Context B), and in another novel context.

(c) Both groups of mice display robust memory linking, such that the freezing in both context A (linked) and context B (shock) is higher than freezing in a novel context (Two-way RM ANOVA, F_{time} (1.9, 54) = 86.9, $p < 0.0001$; Dunnett's multiple comparisons test; n = 16 and 14 mice for Control and cFos-tTa groups).

Data represent mean ± s.e.m. and each data point, *** $p < 0.001$, **** $p < 0.0001$.

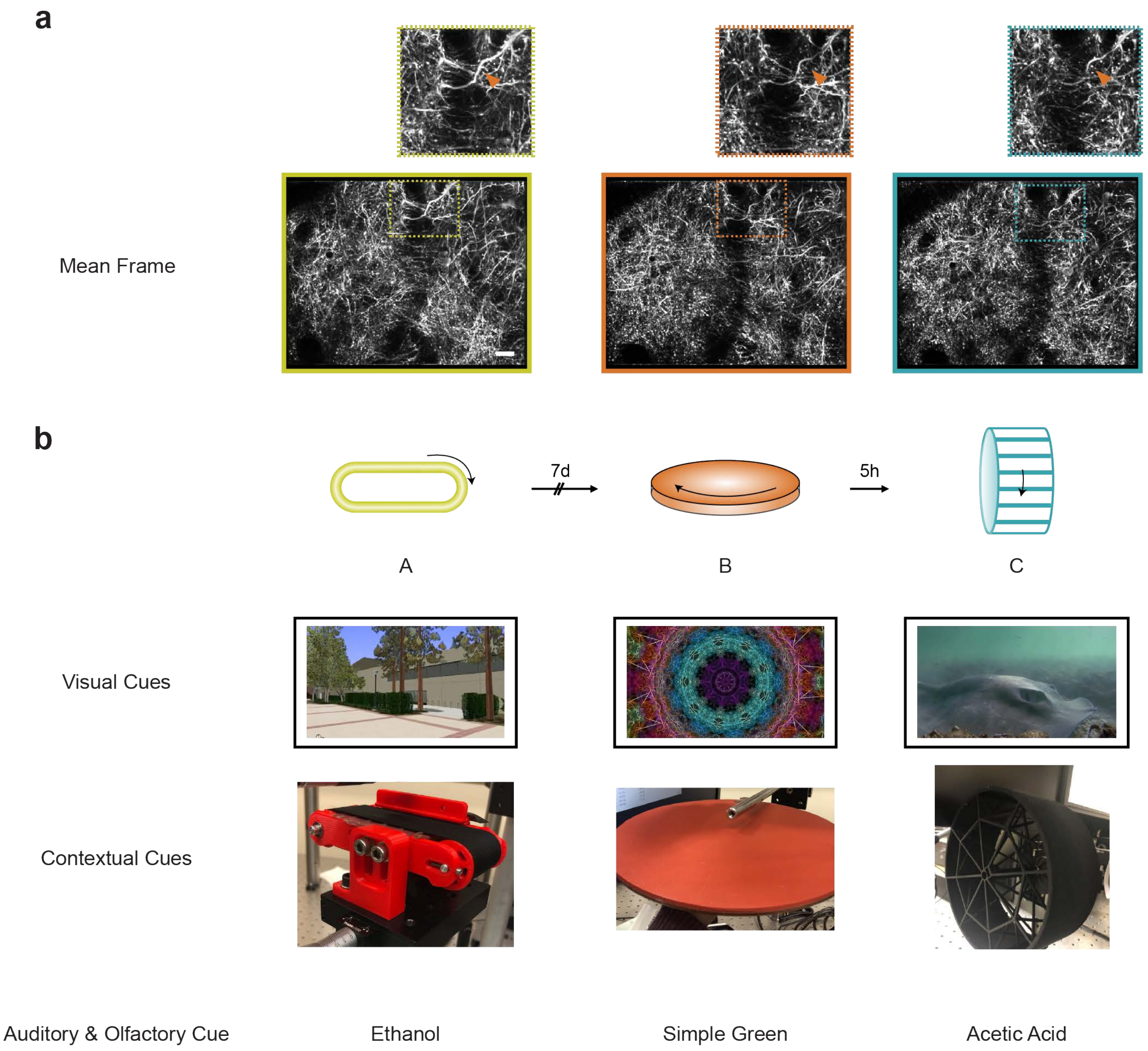
a
Mean Frame
b
7d
5h
A
B
C
Visual Cues
Contextual Cues
Auditory & Olfactory Cue
Ethanol
Simple Green
Acetic Acid

Supplementary Figure 3. Experimental setup for functional dendritic co-allocation studies:

(a) Bottom: Example maximum projection images from three imaging sessions from a mouse. Scale: 20 μm. Top: Inset demonstrates boxed region from each image below magnified to depict the same dendritic segment across sessions.

(b) Schematics of 3 distinct contexts (different auditory, visual, and olfactory cues as well as running apparatus) used in the head-fixed experiments. Mice were exposed to 3 distinct contexts 7 days or 5 hours apart in a counterbalanced manner while RSC dendritic transients were imaged.

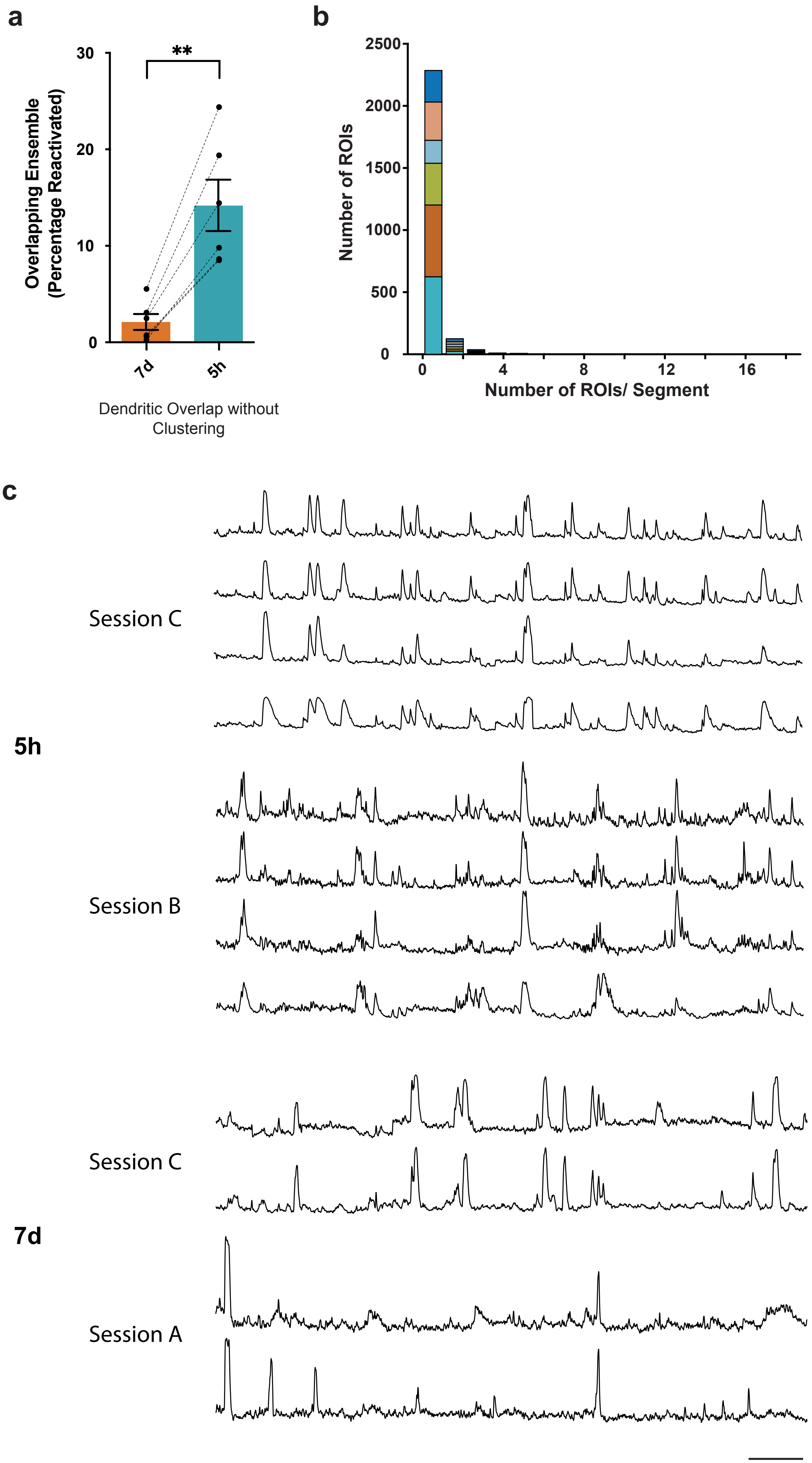

a
b
**
30
20
10
0
Overlapping Ensemble
(Percentage Reactivated)
7d
5h
Dendritic Overlap without Clustering
2500
2000
1500
1000
500
0
Number of ROIs
0
4
8
12
16
Number of ROIs/ Segment
c
Session C
5h
Session B
Session C
7d
Session A

Supplementary Figure 4. Differences in dendritic overlap are not driven by correlated activity within dendritic branches.

(a) Dendritic overlap is higher when context exposures are 5 hours (5h) apart vs. 7 days (7d) apart when dendritic ROIs are not clustered together based on correlated activity. (Paired t-test; $t = 6.5$; $p = 0.001$; n=6 mice). Data represent mean ± s.e.m. and each data point; all comparisons were two-tailed.

(b) Histogram of the number of ROIs per dendritic segment following clustering (1.15 ± .03 ROIs per cluster). Data from each mouse is depicted in a separate color.

(c) For the reactivated dendritic segments (i.e., the clustered ROIs within reactivated segments), ROIs clustered based on their activity in session C (reference session) display high within-cluster correlated activity across sessions. Traces represent z-score of calcium transients. Scale: 30s.

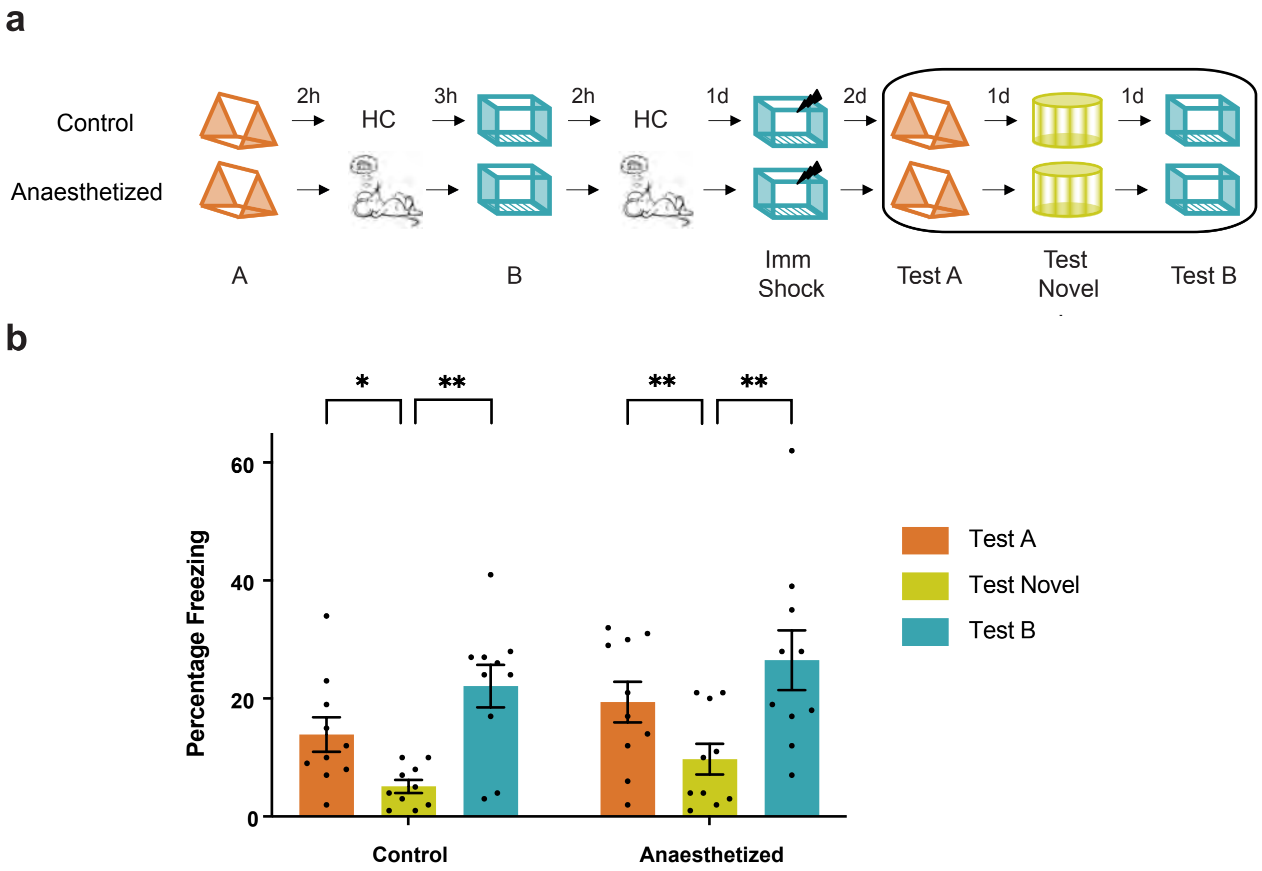

a
Control
Anaesthetized
2h
HC
3h
2h
HC
1d
2d
1d
1d
A
B
Imm
Shock
Test A
Test
Novel
Test B
b
*
**
**
**
Percentage Freezing
60
40
20
0
Control
Anaesthetized
Test A
Test Novel
Test B

Supplementary Figure 5. Memories of contexts can still be linked under conditions used during structural imaging.

(a) Experimental setup: All mice were handled and habituated in a manner identical to the imaging experiments in Figure 4. Mice experienced two contexts (A and B) 5 hours apart. Two hours following each context exposure, mice were anesthetized for 40 minutes to mimic anesthesia during the imaging sessions to study spine dynamics.

(b) The anesthetized mice can link the shock context (context B) to a neutral context (context A) 5 hours apart. For both groups of mice, freezing in the linked (context A), as well as training context (context B), is higher than freezing in a novel context. Therefore, prolonged anesthesia on the day of memory linking does not disrupt memory linking. (Two-way RM ANOVA, $F_{context}$ (1.5, 27.8) = 27.8, $p < 0.0001$, Dunnett's multiple comparisons test; n = 10 each).

Data represent mean± s.e.m. and each data point, * $p < 0.05$, ** $p < 0.01$.

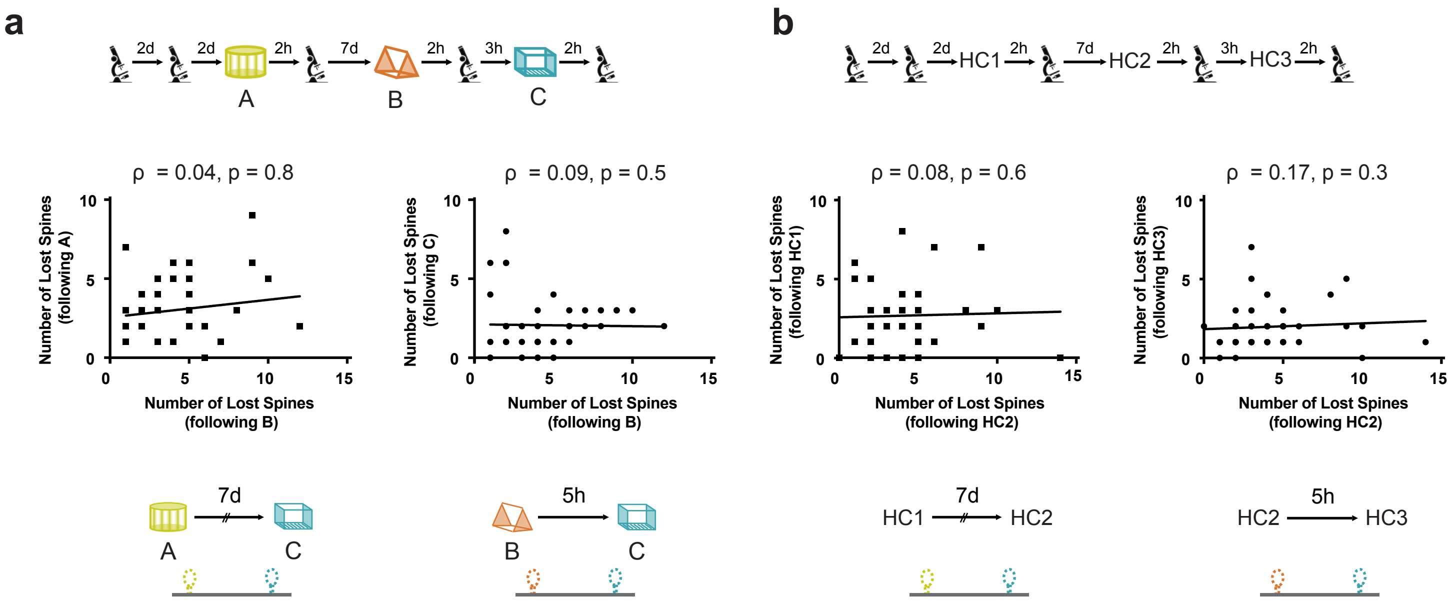

a
2d
2d
2h
7d
2h
3h
2h
A
B
C
ρ = 0.04, p = 0.8
Number of Lost Spines (following A)
Number of Lost Spines (following B)
ρ = 0.09, p = 0.5
Number of Lost Spines (following C)
Number of Lost Spines (following B)
7d
A
C
5h
B
C
b
2d
2d
HC1
2h
7d
HC2
2h
3h
HC3
2h
ρ = 0.08, p = 0.6
Number of Lost Spines (following HC1)
Number of Lost Spines (following HC2)
ρ = 0.17, p = 0.3
Number of Lost Spines (following HC3)
Number of Lost Spines (following HC2)
HC1
7d
HC2
HC2
5h
HC3

Supplementary Figure 6. Spine loss during memory linking is not biased to the same dendritic segments

(a) Left: Number of spines lost from a dendritic segment following Context A and B exposure (7 days apart) are not correlated (ρ = 0.04, p = 0.8). Right: Number of spines lost from a dendritic segment following Context B and C (5 hours apart) exposure are not correlated (ρ = 0.09, p = 0.5).

(b) For mice left in their home cages (HC), the number of spines lost from a dendritic segment are not correlated whether imaging sessions are separated by either 7 days (left, ρ = 0.08, p = 0.6) or 5 hours (right, ρ = 0.17, p = 0.3).

Spearman's correlation was used; all comparisons were two-sided.

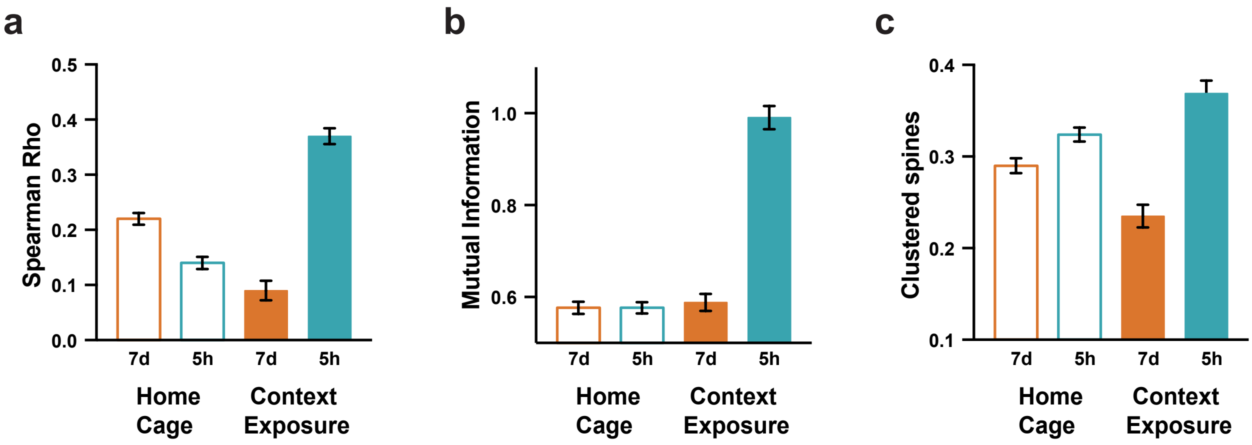

a
b
c
Spearman Rho
0.5
0.4
0.3
0.2
0.1
0.0
Mutual Information
1.0
0.8
0.6
Clustered spines
0.4
0.3
0.2
0.1
7d
5h
7d
5h
Home Cage
Context Exposure

Supplementary Figure 7. Spine dynamics measures following resampling.

Given that resampling distributions in Figure 5e-g are not independent of one another, we plotted the observed values of a) Spearman's rho, b) mutual information, and c) the proportion of clustered spines for each group along with confidence intervals estimated from resampled distributions. Please note that mutual information values are dependent on the number of samples in each group and the present distribution does not control the difference in the number of samples in each group. (Experimental: n = 45 dendrites, 6 mice; Control: n = 42 dendrites, 5 mice). Column graphs represent mean ± 95% confidence intervals; no statistical comparisons were performed.

Figure 2d
Context and fear encoding together (5h apart) to allow optogenetic manipulation 24h post-tagging

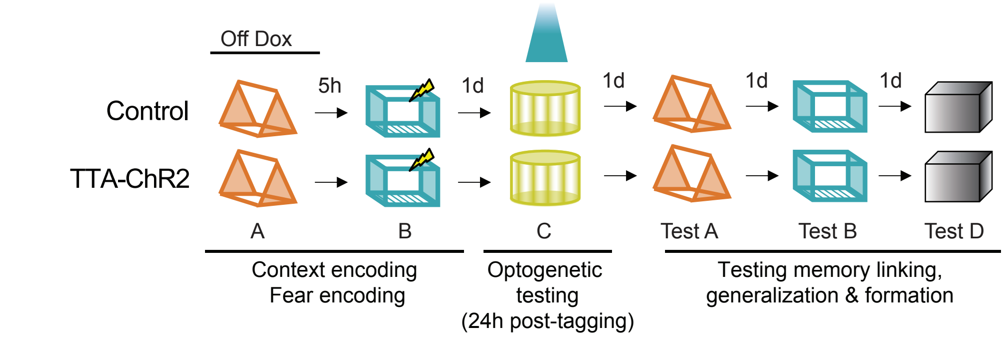

Figure 2e, 6f
Context and fear encoding are separated
Two contexts encodes (2d apart) to prevent linking under control conditions
Optogenetic manipulation 24h post-tagging

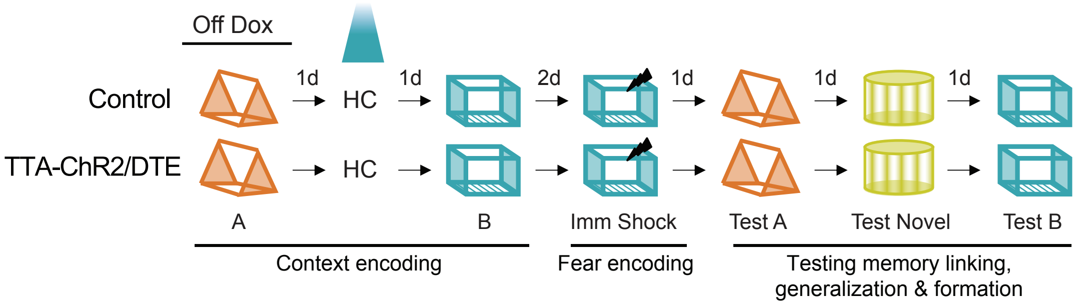

Figure S7
Context and fear encoding are separated
Two contexts encodes (2d apart) to prevent linking under control conditions
Chemogenetic activation 45mins pre-exposure

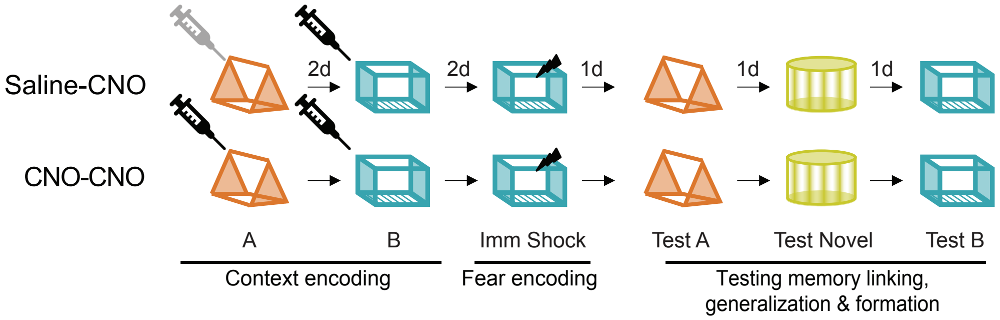

Extended Figure 5
Context and fear encoding are separated
Two contexts are encoded (5h apart) to test the effect of anaesthesia on linking
45mins anaesthesia post-exposure

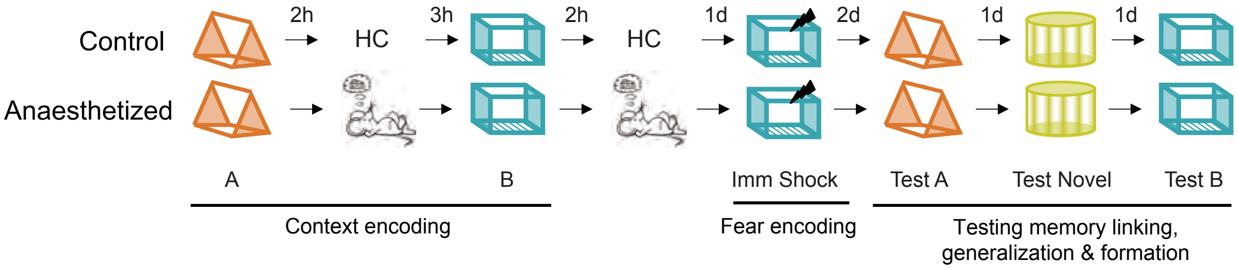

Supplementary Figure 8. Rationale for behavioral experiments investigating memory linking.
