# Supplementary material for: Compartmentalized dendritic plasticity in the mouse retrosplenial cortex links contextual memories formed close in time
Source: Nat Neurosci. 2025 Feb 17;28(3):602–15. doi: 10.1038/s41593-025-01876-8 (PMC11893454; doi:10.1038/s41593-025-01876-8)
Supplement: Supplementary file 2 — Reporting Summary [file 41593_2025_1876_MOESM2_ESM.pdf]

nature portfolio

Corresponding author(s): Alcino J. Silva, Megha Sehgal, Panayiota Poirazi

Last updated by author(s): Nov 25, 2024

Reporting Summary

Nature Portfolio wishes to improve the reproducibility of the work that we publish. This form provides structure for consistency and transparency in reporting. For further information on Nature Portfolio policies, see our Editorial Policies and the Editorial Policy Checklist.

Statistics

For all statistical analyses, confirm that the following items are present in the figure legend, table legend, main text, or Methods section.

n/a	Confirmed	
☐	☒	The exact sample size (n) for each experimental group/condition, given as a discrete number and unit of measurement
☐	☒	A statement on whether measurements were taken from distinct samples or whether the same sample was measured repeatedly
☐	☒	The statistical test(s) used AND whether they are one- or two-sided<br>*Only common tests should be described solely by name; describe more complex techniques in the Methods section.*
☐	☒	A description of all covariates tested
☐	☒	A description of any assumptions or corrections, such as tests of normality and adjustment for multiple comparisons
☐	☒	A full description of the statistical parameters including central tendency (e.g. means) or other basic estimates (e.g. regression coefficient) AND variation (e.g. standard deviation) or associated estimates of uncertainty (e.g. confidence intervals)
☐	☒	For null hypothesis testing, the test statistic (e.g. F, t, r) with confidence intervals, effect sizes, degrees of freedom and P value noted<br>*Give P values as exact values whenever suitable.*
☐	☒	For Bayesian analysis, information on the choice of priors and Markov chain Monte Carlo settings
☐	☒	For hierarchical and complex designs, identification of the appropriate level for tests and full reporting of outcomes
☒	☐	Estimates of effect sizes (e.g. Cohen's d, Pearson's r), indicating how they were calculated

Our web collection on statistics for biologists contains articles on many of the points above.

Software and code

Policy information about availability of computer code

Data collection	We used commercially available softwares for fear conditioning (Video Fear Conditioning "Video Freeze®" Software (Med Associates) and two-photon imaging experiments (Neurolabware Scanbox available at: http://neurolabware.com). For one-photon imaging, open source miniscope software was used (available here: https://github.com/Aharoni-Lab/Miniscope-v4; DAQ software (written in C++ andOpen Computer Vision libraries for mini-scope image acquisition, v0.171). Confocal imaging: NIS-Elements AR (Nikon,v4.40.00), , Electrophysiology: pCLAMP 10 and Digidata®1440A
Data analysis	Customized open source code (CNMFe, Suite2p for 1p and 2p functional imaging respectively was used) and are available from private Github repository: Almeida-FilhoDG/ConcatMiniscope (vl.0.0, DOI: 10.5281/zenodo.5676164) and https://github.com/MouseLand/suite2p . Nikon NIS-Elements AR Analysis (v4.40.00), Matlab R2020b (v9.9.0.1524771), Graphpad Prism6. Fear conditioning data was analyzed using Med associates in built analysis. Immunohistochemistry was analyzed using Imaris 9.0 or NIS-Elements AR Analysis software (v4.40.00). Spine imaging data was analyzed using Scanimage's Spine analysis module.

For manuscripts utilizing custom algorithms or software that are central to the research but not yet described in published literature, software must be made available to editors and reviewers. We strongly encourage code deposition in a community repository (e.g. GitHub). See the Nature Portfolio guidelines for submitting code & software for further information.

Data

Policy information about availability of data

All manuscripts must include a data availability statement. This statement should provide the following information, where applicable:

- Accession codes, unique identifiers, or web links for publicly available datasets
- A description of any restrictions on data availability
- For clinical datasets or third party data, please ensure that the statement adheres to our policy

The original videos and datasets generated during and/or analyzed during the current study are available from the corresponding authors.
We choose to share data on request due to the fact that video recording files are very large and hard to access online. Additionally, our data includes complex and diverse experimental conditions, and our lab can best help people access the data according to their specific requests.

Research involving human participants, their data, or biological material

Policy information about studies with human participants or human data. See also policy information about sex, gender (identity/presentation), and sexual orientation and race, ethnicity and racism.

Reporting on sex and gender	NA
Reporting on race, ethnicity, or other socially relevant groupings	NA
Population characteristics	NA
Recruitment	NA
Ethics oversight	NA

Note that full information on the approval of the study protocol must also be provided in the manuscript.

Field-specific reporting

Please select the one below that is the best fit for your research. If you are not sure, read the appropriate sections before making your selection.

☒ Life sciences ☐ Behavioural & social sciences ☐ Ecological, evolutionary & environmental sciences

For a reference copy of the document with all sections, see nature.com/documents/nr-reporting-summary-flat.pdf

Life sciences study design

All studies must disclose on these points even when the disclosure is negative.

Sample size	We determined sample size based on previous experiments employing similar experimental design (Cai et al., 2016 PMID: 27251287; Shen et al., 2022 PMID: 35614219).
Data exclusions	Patch Clamp experiments: We only included data from cells where the resting membrane potential was > -50mV.
Replication	Experiments were repeated, and the results are reproducible.<br>Briefly, for behavior task, each result was collected from at least 2 independent experiments. Representative histological images were repeated independently in different mice (no less than 3) with similar results.
Randomization	Experiments comprising of genetically wildtype mice: Mice were randomly assigned to groups using a random number generator. In experiments where transgenic animals were used, each genotype was represented during each trial or replication (as appropriate).
Blinding	Behavioral experiments: experimenter was blinded to the genotype, virus injection or drug administered. Imaging and immunohistochemistry: experimenter was blinded to group identity prior to analysis and when possible during experimentation. Patch clamp electrophysiology: experimenter was blinded experimental group (or virus injection). Drug administration: experimenter was blinded to the vehicle/drug aliquot identity.

Reporting for specific materials, systems and methods

We require information from authors about some types of materials, experimental systems and methods used in many studies. Here, indicate whether each material, system or method listed is relevant to your study. If you are not sure if a list item applies to your research, read the appropriate section before selecting a response.

Materials & experimental systems

n/a	Involved in the study	
☐	☒	Antibodies
☒	☐	Eukaryotic cell lines
☒	☐	Palaeontology and archaeology
☐	☒	Animals and other organisms
☒	☐	Clinical data
☒	☐	Dual use research of concern
☐	☐	Plants

Methods

n/a	Involved in the study	
☒	☐	ChIP-seq
☒	☐	Flow cytometry
☒	☐	MRI-based neuroimaging

Antibodies

Antibodies used

guinea pig anti-RFP: SySy 390 004; Secondary Antibodies: goat anti-guinea pig Alexa Fluor 568: Invitrogen (A11075); chicken anti-RFP: SySy 409006, anti-PSD95: SySy 124308, anti-phospho-Cofilin: Millipore C8992; Goat anti-Guinea Pig IgG Alexa Fluor™ 488, Catalog #A-11073; Goat anti-Rabbit Alexa Fluor™ 647, Catalog # A-21245, Goat anti-Chicken, Alexa Fluor™ 594, Catalog # A-11042

Validation

https://www.sysy.com/product-factsheet/SySy_390004, Amygdala inhibitory neurons as loci for translation in emotional memories. Shrestha P, Shan Z, Mamcarz M, Ruiz KSA, Zerihoun AT, Juan CY, Herrero-Vidal PM, Pelletier J, Heintz N, Klann E Nature (2020) 5867829: 407-411. . IHC; tested species: mouse
Astrocyte-neuron subproteomes and obsessive-compulsive disorder mechanisms.
Soto JS, Jami-Alahmadi Y, Chacon J, Moye SL, Diaz-Castro B, Wohlschlegel JA, Khakh BS Nature (2023) : . . IHC; tested species: mouse
https://sysy.com/product/409006, Recognizes mScarlet, mRFP, mCherry and tdTomato. Endocytosis in the axon initial segment maintains neuronal polarity.
Eichel K, Uenaka T, Belapurkar V, Lu R, Cheng S, Pak JS, Taylor CA, Südhof TC, Malenka R, Wernig M, Özkan E, et al. Nature (2022) : . . ICC; tested species: rat

https://www.sigmaaldrich.com/US/en/product/sigma/c8992
Regulation of actin dynamics through phosphorylation of cofilin by LIM-kinase.
S Arber et al.
Nature, 393(6687), 805-809 (1998-07-09)

https://www.sysy.com/product-factsheet/SySy_124308
Microglial Rac1 is essential for experience-dependent brain plasticity and cognitive performance.
Socodato R, Almeida TO, Portugal CC, Santos ECS, Tedim-Moreira J, Galvão-Ferreira J, Canedo T, Baptista FI, Magalhães A, Ambrósio AF, Brakebusch C, et al.
Cell reports (2023) 4212: 113447. 124 308 IHC; tested species: mouse

Animals and other research organisms

Policy information about studies involving animals; ARRIVE guidelines recommended for reporting animal research, and Sex and Gender in Research

Laboratory animals

Male and female C57BL/6 mice acquired from Taconic or Jackson laboratories. Male and female transgenic mice (Thy1-YFP; Jackson Laboratories, Stock No: 00378) bred on C57BL/6 Jackson backgrounds (2 months - 8 months) were used as described in the methods. cFos-tTa mice (gift from Mark Mayford) were maintained on C57BL/6 Taconic background.
Mice are housed in AAALAC accredited facility with 12-12 light/dark cycles. Housing conforms to The Guide for the Care and Use of Laboratory Animals, th edition. The temperature set point is 72 degrees plus or minus 3 degrees; the humidity range is between 30-70%.

Wild animals

No wild animals were used in the study

Reporting on sex

Our findings apply to both sexes. All main findings were replicated in both sexes and data were pooled for final analysis and figures. We did not observe any sex-specific differences in our findings. Sex was determined using anogenital distance.

Field-collected samples

No field collected samples were used in the study

Ethics oversight

Chancellor's Animal Research Committee of the University of California, Los Angeles

Note that full information on the approval of the study protocol must also be provided in the manuscript.

Plants

Seed stocks	NA
Novel plant genotypes	NA
Authentication	NA
